# Sub-nanometer 3D morphometric precision of polarisation-resolved wide-field optical extinction microscopy determines the roundness of individual gold nanospheres

**DOI:** 10.1039/d6nr03059a

**Published:** 2026-09-15

**Authors:** Lukas M. Payne, Furqan Alabdullah, Paola Borri, Wolfgang Langbein

**Affiliations:** a School of Physics and Astronomy, Cardiff University The Parade Cardiff CF24 3AA UK; b School of Biosciences, Cardiff University Museum Avenue Cardiff CF10 3AX UK; c School of Engineering, Cardiff University The Parade Cardiff CF24 3AA UK; d Engineering Technical College of Al-Najaf, Al-Furat Al-Awsat Technical University Najaf 31001 Iraq; e Cardiff University School of Physics and Astronomy The Parade Cardiff CF24 3AA UK langbeinww@cardiff.ac.uk

## Abstract

Quantitative polarisation-resolved optical extinction microscopy of individual plasmonic nanoparticles has recently been introduced as a powerful approach to characterise the nanoparticle's morphology with a precision comparable to that of electron microscopy, while using a simple optical microscope [L. M. Payne, W. Albrecht, W. Langbein and P. Borri, *Nanoscale*, 2020, **12**, 16215–16228]. Here we provide a step change by adding measurements for radial polarisation in the condenser back focal plane, probing plasmonic resonances polarised in the axial direction. The combined linear and radial polarisation measurements provide a significantly enhanced precision of the retrieved 3D morphology, as we show on defect-free ultra-uniform gold nanospheres of 30 nm nominal diameter characterised by transmission electron microscopy. The measured cross-sections are modelled for an ellipsoidal particle, determining the three semi-axes and rotation angles by fitting the measurements. The material permittivity and surface damping providing the best fit are found. The particle aspect ratio is determined with a precision better than 5%, and the size with an impressive precision of 0.1 nm. Notably, corrections to the Rayleigh–Gans ellipsoid model due to retardation are significant even though the particle diameters are more than an order of magnitude smaller than the wavelength in the medium, and taking them into account reduces the systematic error in the retrieved diameters from 2 nm to below 1 nm. The accuracy of size and shape determination is limited by the remaining systematic errors in modelling.

## Abstract 2

Introducing radially polarized light and considering retardation enables the nanosizer to achieve a precision of 0.1 nm in size and a few % in shape, for high-throughput morphometric studies of nanoparticles within a known shape and material class.

## Graphical abstract

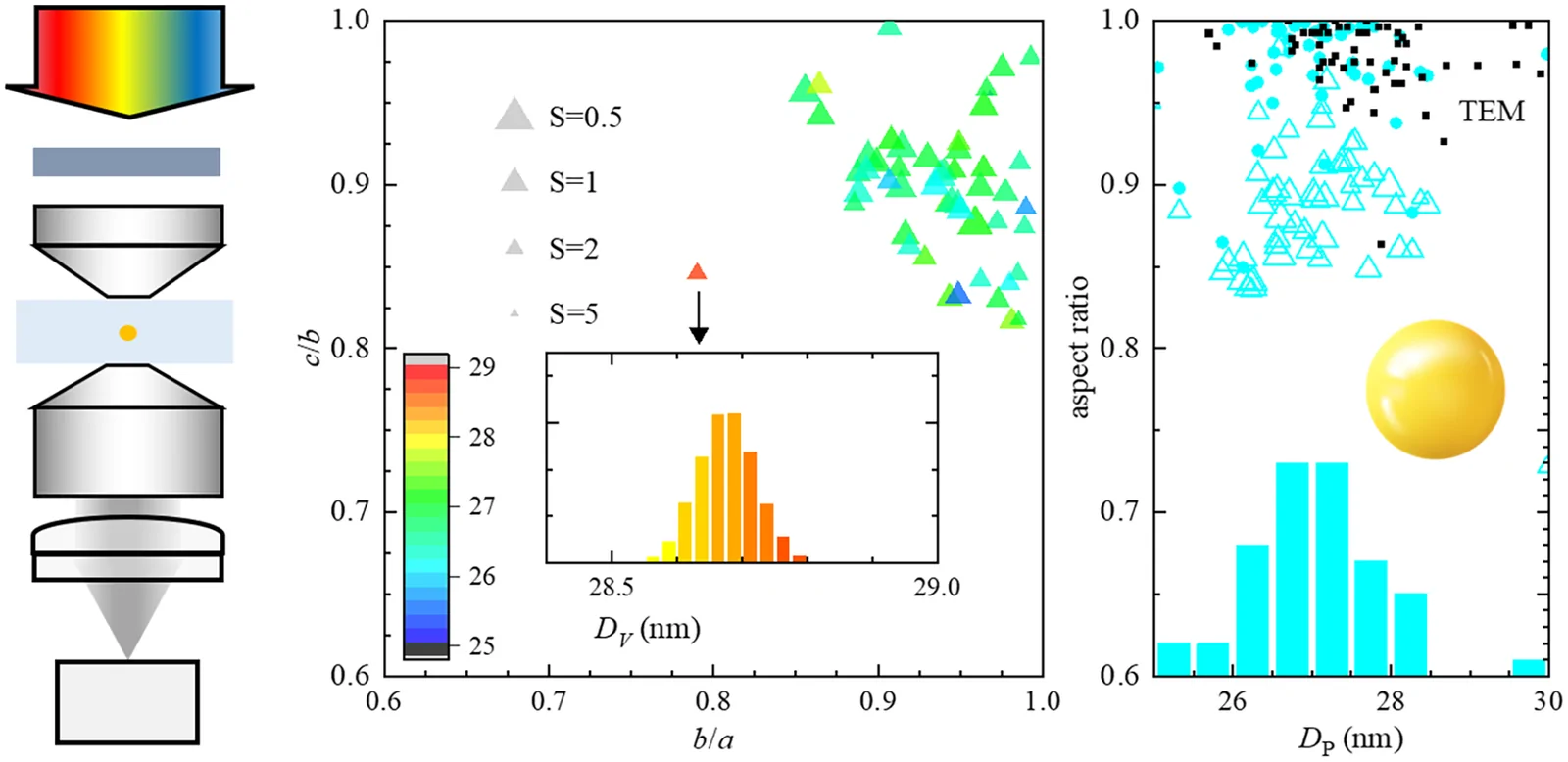

## I. Introduction

Over the last few decades, nanoparticles (NPs) and nanostructured materials have become increasingly important in many fields, from biological and medical imaging,^1,2^ to sensing,^3,4^ catalysis,^5,6^ and drug-delivery.^7,8^ Considering the plethora of nano-objects produced by human activity, studies of NP waste and environmental contamination^9^ have attracted increasing attention. Throughout these spaces, the size and shape of NPs play key roles, hence their accurate characterization is important.^10^

For NPs larger than the diffraction limit of visible light of around 250 nm, optical microscopy can directly resolve the NP morphology. However, for smaller NPs such a characterization remains a significant challenge. To date, the industry standard for NP morphometry remains transmission electron microscopy (TEM),^10,11^ but due to high capital and operating costs, TEM is often not economically viable. Additionally, TEM is time-consuming, specifically if tomography is needed for 3D analysis, limiting the number of NPs measured and thus the statistical relevance of the results. Furthermore, the high energy electron beam used can lead to NP damage/reshaping during measurements. These aspects hinder the applicability of TEM to high-throughput and reliable statistical assessment of samples, a critical feature of quality control in NP manufacturing.

Apart from microscopy, in recent years techniques exploiting NP mechanical properties have shown promise in NP sizing, such as centrifugation,^12,13^ field-flow fractionation (FFF) in various implementations,^13–15^ and tunable resistive pulse sensing (TRPS).^13,16,17^ Various versions are commercially available as benchtop devices, but these techniques also exhibit limitations. Centrifugation methods yield ensemble properties of particles only, and all methods provide size but not shape metrics, except FFF which provides indirect shape information.

Popular optical methods for NP morphometry use the light scattered from an incident field by NPs in suspension, to monitor their motion in a viscous liquid medium. These methods include dynamic light scattering (DLS),^18^ nanoparticle tracking analysis (NTA),^19,20^ and interferometric NTA (iNTA).^21^ Both DLS and NTA are implemented in commercially available benchtop devices, with DLS being an industrial standard for rapid sample size characterization. DLS is an ensemble measurement technique, strongly affected by sample polydispersity, while NTA and iNTA both provide single particle measurements, overcoming this issue. However, all three assume a spherical particle shape undergoing Brownian motion to determine the hydrodynamic radius, which is in itself distinct from the physical radius. Hence, these methods provide no shape information, and use the optical measurements only indirectly to determine the NP size. A nanofluidic scattering-based technique also exists, but is presently restricted to determining the hydrodynamic radius as well.^22^ Alternatively, depolarised dynamic light scattering (DDLS) leverages the detection of the co- and cross-polarised components of the scattering signal to measure the translational and rotational diffusion coefficients, and subsequently the length and diameter of the particles assuming a rod or cylinder-like particle model. However, being a DLS-based technique, it still lacks individual particle measurements, and the determined values are reflective of the apparent size from NP motion through liquid. Other recent advances in photopolarimetric techniques without^23^ and with^24^ structured illumination exhibit similar limitations. Additional methods include through-focus scanning optical microscopy (TSOM)^25^ and through-focus interferometric nanoparticle imaging^26,27^ which use the measured signal from particles at different focal positions.

Complementary approaches directly measure optical properties for diffraction-limited NP morphometry, using either the complex polarisability,^28,29^ or the optical cross-sections, namely absorption (*σ*_abs_),^30–32^ scattering (*σ*_sca_),^33–35^ and extinction (*σ*_ext_).^31,34,36,37^*σ*_abs_ and *σ*_sca_ are fundamentally related to the NP polarisability, and quantify the ability of an object to absorb or scatter light, respectively. The extinction cross-section, *σ*_ext_, is the sum of the absorption and scattering, and is given by the effective area blocked by the particle for a collimated incident field. These optical methods may be employed in conjunction with a physical model, to compare theoretical predictions of morphometry-dependent optical properties with optical measurements, and then infer the NP size and shape, frequently referred to as ‘solving the inverse problem’. The polarisability and the cross-sections of NPs of given size and shape can be calculated using analytical models only in special cases, such as spheres,^38^ or ellipsoids within the dipole approximation,^39^ while for more complex shapes numerical models are needed.^40–42^

Recently, our group established a methodology for 3D morphometry of single NPs called the Optical Nanosizer (ON).^43^ As a wide-field microscopy modality, the ON offers simultaneous measurement of up to hundreds of NPs in a single field of view, currently with a sensitivity down to *σ*_ext_ ≈ 1 nm^2^, corresponding to the cross-section of a gold nanosphere (GNS) with a diameter *D* ≈ 2 nm. In this method, a series of brightfield transmission images of NPs deposited on a coverslip are captured with a conventional microscope under illumination at different wavelengths and linear polarisations. Dark-field images can be additionally taken to distinguish scattering and absorption cross-sections.^35^ Images are analysed as differential transmission contrast, to quantify *σ*_ext_ for each NP in the field of view. By comparing the Rayleigh–Gans model for ellipsoidal particles with the measurements of *σ*_ext_ for each NP in the images, we inferred the size and shape of nominally spherical small (10 to 30 nm diameter) gold nanoparticles, and gold nanorods (10 nm diameter, 30 nm length). The precision of the method was found to be ±0.5 nm for size, and about 10% for the shape anisotropy. In terms of accuracy, we found the measurements of GNS having a nominal diameter, *D* = 30 nm, to be within 1 nm of the TEM characterization result on the same sample, *i.e.* showing only about 4% discrepancy. Notably, for shape retrieval we used linearly polarised excitation light in the condenser back focal plane with varying in-plane angles. Even with a high numerical aperture (NA) condenser focussing the light, only a small fraction of light at the sample is polarised out of plane, *i.e.* axially. Hence, the measurements are dominated by excitation polarisations in the sample plane, and thus are more sensitive to the in-plane geometry of the NP. The out-of-plane geometry was therefore indirectly retrieved from spectral properties using the dependence of surface plasmon resonance frequencies on particle morphology.

In this work, we present an ON with 3D shape recovery independent of orientation, by using both linear and radially polarised illumination. In conjunction with a high NA condenser, radially polarised light creates a strong axial polarisation component at the sample. This provides a direct probe of the out-of-plane NP geometry, while the linear in-plane polarised illumination probes the in-plane NP geometry. We examine nominal *D* = 30 nm ‘ultra-uniform’ gold nanospheres (UGNS) to demonstrate the ON capabilities. Notably, these UGNS had been characterized with high-angle annular dark-field scanning transmission electron microscopy (HAADF-STEM) in our previous works,^43,44^ including tomography on selected cases, providing a reliable morphometry ground truth. The new ON method shows a significant improvement in the precision and accuracy of 3D size and shape retrieval, reaching impressive sub-nm values.

## II. Model: permittivity, polarisability, and optical cross-sections

The previous ON analytical developments are described in detail in ref. 43 and 44. We build here on this analysis, extending it to add the treatment of the radially polarised illumination. We briefly recap the model in the following. The morphometry of each particle is derived from the measured cross-sections by solving ‘the inverse problem’, using a theoretical framework relating the geometry of a NP to its optical cross-sections. Modelling the optical cross-sections requires knowledge of the complex permittivity of the NP material. In the literature, different measured spectra of the permittivity of gold are provided. We considered three of these, namely the data of Johnson & Christy (JC),^45^ McPeak *et al.* (MP),^46^ and Olmon *et al.* (OL).^47^ They were measured by spectroscopic ellipsometry on thin polycrystalline films (JC and MP) or single crystalline gold (OL). To account for a surface damping effect in the NPs, we use the following approach. The permittivity is described by a Drude model with a bound electron contribution stemming from the interband transitions from the *d* bands into the conduction band, and surface damping effects, given by^35,48^1
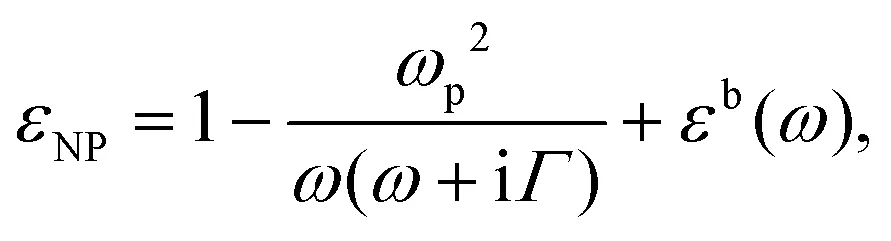
with *ω*_p_ being the plasma frequency, *ε*^b^ the bound electron contribution,^48,49^ and *Γ* the Drude damping rate. A surface damping term is included in *Γ*, which is approximated^50^ by2
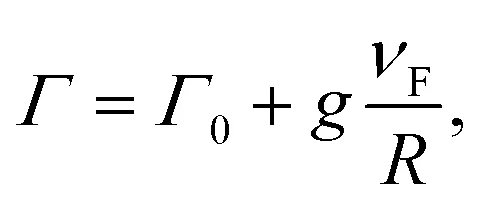
with the NP radius, *R* = *D*/2, the bulk damping rate, *Γ*_0_, the Fermi velocity in gold,^51^*v*_F_ = 1.4 × 10^6^ m s^−1^, and a dimensionless number, *g*, parameterizing the surface damping – Table S1 of ref. 35 provides the complete parameterization including *ε*^b^(*ω*). Assuming *g* = 0, we determine the values of all the other parameters by using eqn (1) to independently fit each experimental gold permittivity dataset. Using these parameters, we determine a series of *ε*_NP_ evaluated with different values of *g* ∈ [0,4.5], covering the range of *g* reported in the literature.^37,48^ We note that the surface damping parameter depends on the surface chemistry, such as functionalisation or coating.^52,53^

We use the Rayleigh–Gans model, describing ellipsoidal NPs much smaller than the light wavelength *λ*, to determine the NP polarisability using the complex material permittivity and geometry of the NP, and the permittivity, *ε*_m_, of the surrounding medium. As introduced in ref. 44, we use a Cartesian reference system of unit vectors 
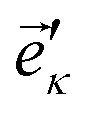
, with *κ* = *x*′,*y*′, and *z*′ which respectively point in the directions of the three semi-axes of an ellipsoidal NP of lengths *a*, *b*, and *c*, which we order such that *a* ≥ *b* ≥ *c*. In this basis, the polarisability tensor *α*′ is a diagonal matrix. The diagonal elements are the polarisabilities for fields oriented along each of the NP's semi-axes, given by3
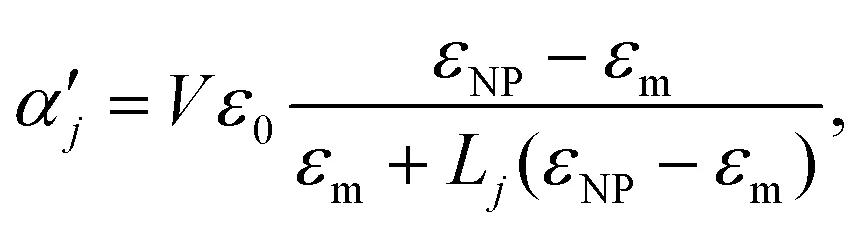
where *V* is the NP volume, *ε*_0_ is the free space permittivity, *ε*_m_ is the dielectric function of the surrounding medium, and *L*_*j*_ is a depolarisation factor, with *j* ∈ [1,2,3]. The geometry of the NP determines *L*_*j*_. For example, for a sphere, *L*_*j*_ = 1/3. *ε*_m_ is assumed to be real and frequency independent. All materials are assumed to be non-magnetic, *i.e.* with relative permeability *μ*_r_ = 1. We note that metals exhibit a zero crossing *versus* wavelength of the real part of the denominator in eqn (3) at 
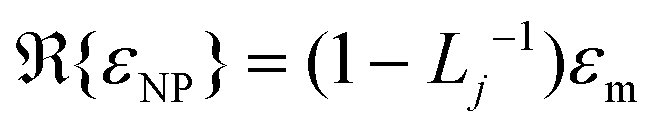
, which results in a peak in *σ*_ext_ called the localised surface plasmon resonance (LSPR). For an incident field 
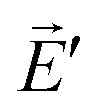
, the induced electric dipole moment is given by4
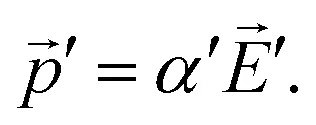


We choose a Cartesian coordinate system to describe the laboratory frame, with axes *ι* ∈ {*x*,*y*,*z*} and associated unit vectors 
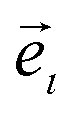
, where 
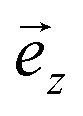
 points along the optical path, and 
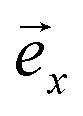
 and 
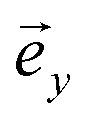
 span the sample plane. To transform *α*′ into the laboratory reference frame, we define^43^ a 3D rotation matrix using *R* = *R*_*ψ*_*R*_*θ*_*R*_*ϕ*_ and *R*^T^ = *R*_*ϕ*_^T^*R*_*θ*_^T^*R*_*ψ*_^T^, with *ϕ*, *θ*, and *ψ* being the angles of rotation about 
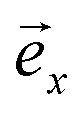
, 
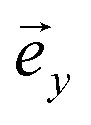
, and 
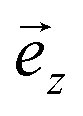
, respectively. We transform a vector from the NP frame into the laboratory frame with 
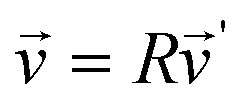
. Eqn (4) becomes 
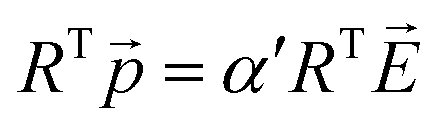
, such that the polarisability in the laboratory frame is given by

5 *α* = *Rα*′*R*^T^.

The scattering and absorption cross-sections, *σ*_sca_ and *σ*_abs_, are the power scattered (*P*_sca_) or absorbed (*P*_abs_) from the incident field by the NP relative to the intensity, *I*_inc_, of the incident field, *σ*_sca_ = *P*_sca_/*I*_inc_ and *σ*_abs_ = *P*_abs_/*I*_inc_. Within the dipole approximation *D* ≪ *λ* the optical cross-sections are given by6
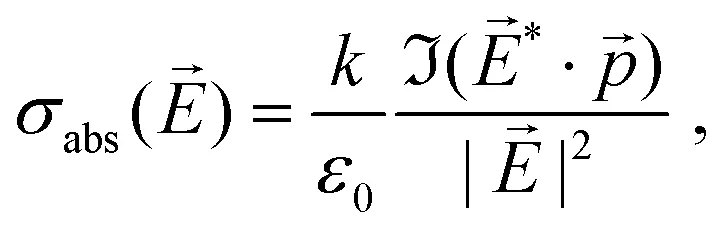
and7
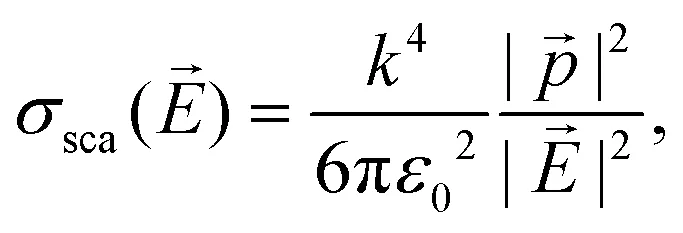
with (*) indicating complex conjugation, and *k* = 2π*n*_m_/*λ*, the wavenumber in the medium of refractive index 
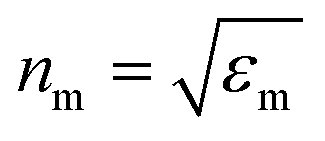
.

To compare the ellipsoid geometry with plane-view TEM characterisation, we define a mean aspect ratio (MAR), 
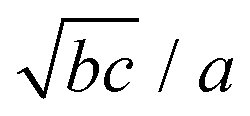
, and a projected aspect ratio (PAR) given by *b*′/*a*′, with the semi-axes lengths *a*′ and *b*′ in the *xy*-plane of the ellipsoid projected along the *z* axis (for calculation details see section S2). We also define the corresponding projected diameter as 
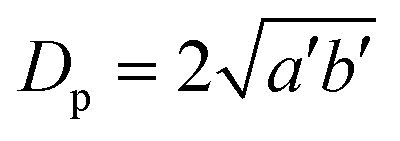
, and a volume-equivalent diameter as 
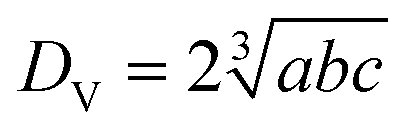
.

## III. Experiment

A detailed description of the microscope setup and data analysis is provided in ref. 43. A brief overview of the set-up and samples used in this work is given in the following sections.

### A. Optical measurements

We performed wide-field optical transmission measurements on a Nikon Ti-U inverted microscope, with illumination provided by a 100 W tungsten halogen lamp, using bandpass filters of 40 nm width and center wavelengths *Λ* ∈ {450 nm, 500 nm, 550 nm, 600 nm, 650 nm, 700 nm}. A 1.34 NA oil-immersion condenser (Nikon MEL41410) focused the illumination to the sample. The transmitted light was collected by a 1.45 NA 100× oil immersion objective (Nikon MRD01905) and imaged by a 1.0× tube-lens onto the camera with a magnification of *M* = 100. A motorised linear polariser in the collimated light path towards the condenser controlled the excitation polarisation angle *γ*_P_, and a motorised slider switched between linear and radial polariser.^54^ A scientific-CMOS (sCMOS) camera (PCO Edge 5.5) at the eyepiece port was used for image acquisition, with 2560 × 2160 pixels and a full-well capacity of *N*_fw_ = 30 000 electrons. Illumination intensities and exposure times were chosen to result in pixel values around 50 000 counts of the 16-bit digitizer range, which for the gain of 0.54 electrons per count corresponds to about 27 000 electrons, close to *N*_fw_ without entering a non-linear response range. For each wavelength, taken in ascending order, the sample was focussed, and then data were taken for linear polarisation at *γ*_P_ values of 0, 45, 90 and 135 degrees, and radial polarisation. A sketch of the microscope set-up and sample geometry is shown in Fig. 1, and more details are given in ref. 54.

**Fig. 1:**
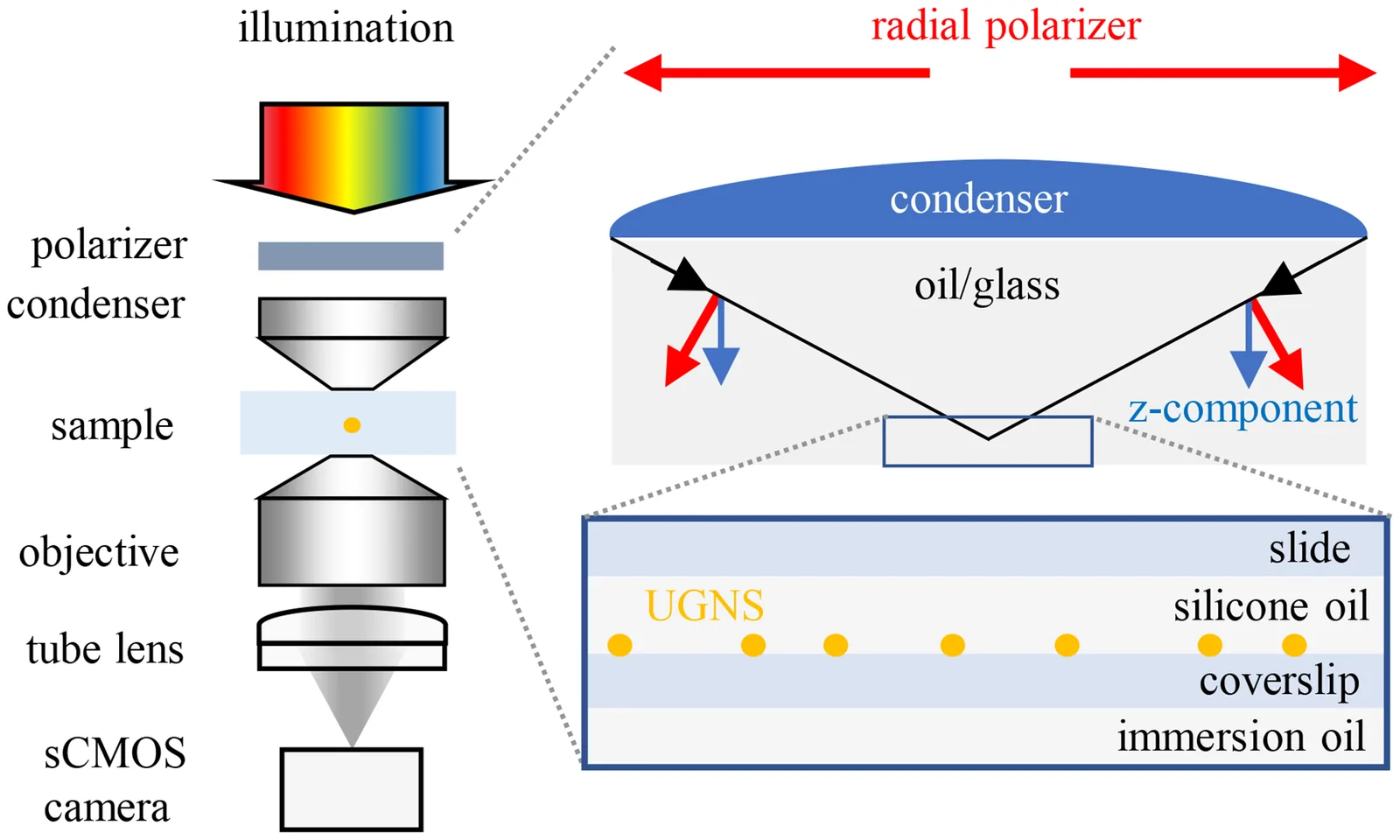
Sketch of the microscope set-up and investigated sample. Under Köhler illumination of a selectable wavelength range, the sample is imaged onto a camera, using a 1.34 NA condenser and a 1.45 NA objective. In the illumination a rotatable linear polariser or a radial polariser is used, the latter generating a significant axially polarised illumination component in the focal plane. The sample consists of individual gold nanospheres deposited onto a glass coverslip, surrounded by silicone oil index-matched to glass.

### B. Samples

‘Ultra-uniform’ gold NPs of nominal spherical shape and mean diameter, *D* = 30 nm, were obtained from NanoComposix. To observe small gold NPs with extinction microscopy, glass slides and coverslips (Menzel-Gläser, #1.5) have to be optically clear, with no contamination or debris. This was achieved using a cleaning process involving sonication steps in toluene and acetone, water rinse followed by treatment with boiling deionised (DI) water, and then storage in 30% hydrogen peroxide for at least one day before preparation. A wet casting method was used for NP deposition, to prevent surface tension-induced aggregation during the sedimentation of NPs on the coverslips in a dry environment. The concentration of NPs in the suspension applied onto the glass surface was adjusted to achieve a surface density with individual NPs separated by a few microns. The stock concentration provided by the manufacturer was diluted accordingly. Slides and coverslips were rinsed with DI water, dried in a nitrogen flow, and 50 µL of the diluted NP suspension was evenly spread across the whole coverslip surface. The sample was placed in a 100% humidity environment for one hour, to allow NP sedimentation *via* wet casting. Afterwards, the coverslip was gently rinsed with DI water, positioned vertically to allow excess water to drain, and dried in a nitrogen flow. Then, 25 µL of silicone oil (AP150, Wacker), index-matched to glass, was pipetted onto the coverslip to create an index-matched medium surrounding the NPs. A glass slide was placed over the oil-covered surface, and the coverslip was pressed manually to reduce the oil layer thickness. Excess oil at the edges of the coverslip was removed and the sample was sealed by applying nail varnish to the edges of the coverslip.

### C. Optical extinction measurements

In ref. 55 we detailed our procedure to quantitatively measure *σ*_ext_, with additional information given in ref. 56. In short, two brightfield images called *I*_1_ and *I*_2_ are captured, which differ only by a lateral shift of the sample, typically 1 to 2 µm (*i.e.* a few optical resolutions). *I*_1_ and *I*_2_ are averaged in real time over a number, *N*_i_, of individual acquisitions to reduce shot noise. We compute *Δ*_1,2_ = 1 − *I*_1,2_/*I*_2,1_ to yield two extinction images (with inverted contrast). To further reduce shot noise and systematic noise due to sensor electronic drift, we can average the extinction over a number of repetitions *N*_r_. For a NP centered in the areas *A*_1,2_ of radius *r*_i_ in *I*_1,2_, we determine its extinction as 
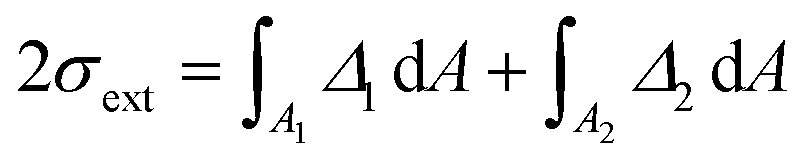
. We calculate the shot-noise-limited standard deviation in the measured *σ*_ext_ as^55^8
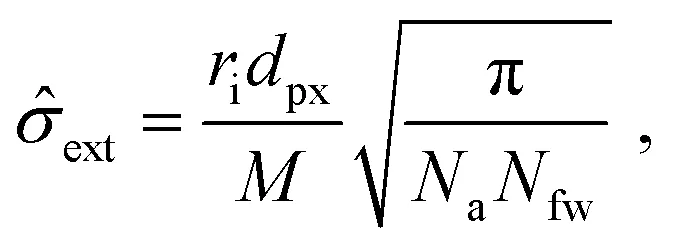
where *M* is the magnification from the sample to the detector, *N*_a_ = *N*_i_*N*_r_ is the number of acquired frames, and *d*_px_ = 6.5 µm is the pixel pitch of the sensor. We previously used^34^ a measurement radius of *r*_i_ = 3*λ*/(2NA) to analyse *σ*_ext_, and the effect of *r*_i_ on *σ*_ext_ is characterised in detail in ref. 57. In the present work, we employ three values of *r*_i_, specifically *r*_s_ = 3*λ*/(2NA), *r*_h_ = 1.2*r*_s_, and *r*_l_ = 0.8*r*_s_, with *N*_i_ = 128 and *N*_r_ = 20. At *Λ* = 550 nm, eqn (8) yields 
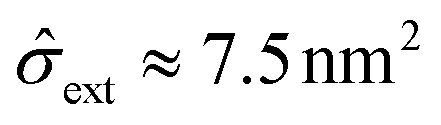
 using *r*_s_, while the noise seen in the extinction data at this wavelength is 15.6 nm^2^. The additional noise is attributed to surface roughness, debris, and/or residual sensor fluctuations, as analysed in detail in ref. 55. We note that measurements were taken over several different fields of view (FOV) and aggregated for the final statistics. The 
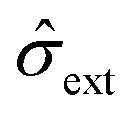
 reported is the polarisation-averaged value of the noise, averaged over the measured values from all the FOVs. We found that 
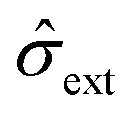
 varies by ±5% across the illumination polarisations, and by ±15% across FOVs. We measure *σ*_ext_ for multiple wavelengths *Λ*, both for the linear polariser at multiple angles *γ*_P_, denoted as *σ*_*Λ*_(*γ*_P_), and for the radial polariser, denoted as *σ*_*Λ*_(*γ*_R_). The noise 
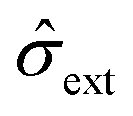
 refers, in general, to the value specific to the data of each *Λ*, polarisation, and field-of-view (see section III A for *Λ* and *γ*_P_ values used). The wavelength dependence of the noise is shown in the inset of Fig. 2a.

**Fig. 2:**
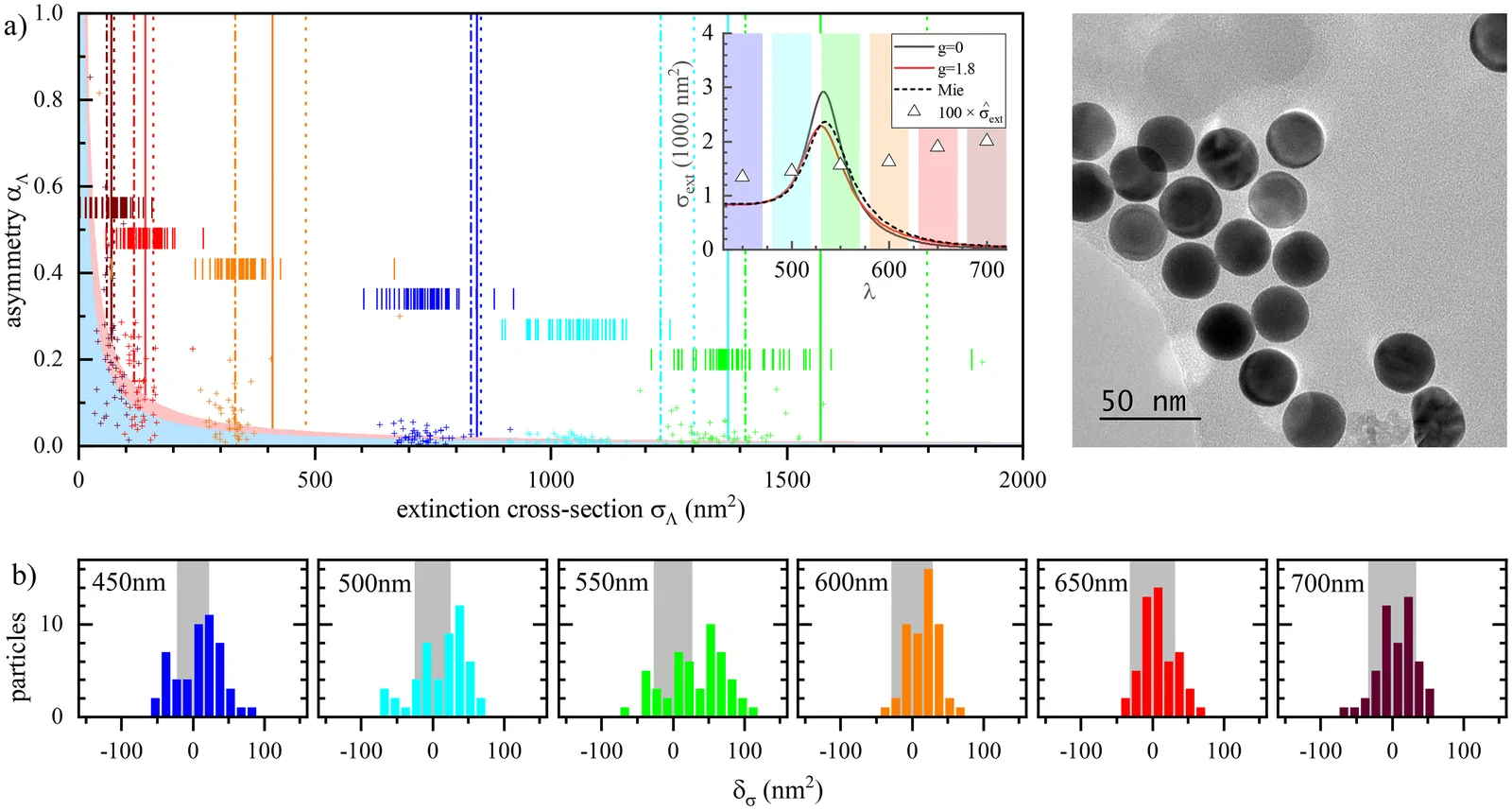
Measured properties of *N* = 51 UGNSs of *D* = 30 nm nominal diameter. (a) Asymmetry *α*_*Λ*_*versus* cross-section *σ*_*Λ*_ for *Λ* = {450 nm, 500 nm, 550 nm, 600 nm, 650 nm, 700 nm}, color-coded to reflect the center wavelength. Coloured crosses indicate the fitted parameters for linear polarisations using eqn (9). Coloured vertical bars indicate the measured *σ*_*Λ*_(*γ*_R_) for radial polarisation, at vertical positions chosen for clarity. A representative TEM image^43,44^ of NPs from the same batch is shown on the right. The noise in *α*_*Λ*_ is shown as shaded areas for *Λ* = 450 nm (bluish) and *Λ* = 700 nm (reddish), estimated as 
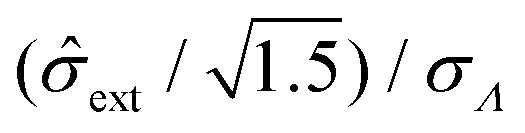
, corresponding to the lowest and highest noise *versus Λ*, respectively. See section S3 for further details regarding the noise in the fit parameters. Solid vertical lines indicate the calculated *σ*_*Λ*_ for a spherical gold NP for each *Λ*, taking into account averaging over filter ranges, for the sample-averaged TEM diameter *D*_V_, 27.94 nm, and the OL permittivity dataset with *g* = 1.8. The corresponding spectrum is given in the inset, showing additionally the case with *g* = 0, and the result of Mie theory which includes retardation, as labelled. Dotted vertical lines indicate the same as solid lines, but calculated using Mie theory. Dashed–dotted lines indicate the same as dotted lines, but for MP permittivity. The corresponding spectrum is shown by the dotted curve in the inset. Vertical colour bands indicate the filter ranges used in the experiment. The measurement errors 
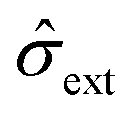
, polarisation-averaged and FOV-averaged, are shown for each *Λ*, scaled by a factor 100. (b) Histograms of *δ*_*σ*_, for each *Λ* as labelled, with gray bands showing 
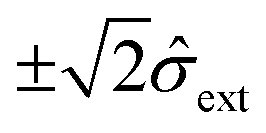
, where the 
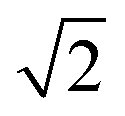
 factor accounts for the additional noise arising from the subtraction in *δ*_*σ*_.

We model the polarisation dependence of *σ*_ext_ for linear polarisation as

9 *σ*(*γ*_P_) = *σ*{1 + *α* cos[2(*γ*_P_ − *γ*)]}

where *σ* is the average cross-section, and the polarisation dependence is given by a relative amplitude *α*∈[0,1], which encodes the dipolar in-plane asymmetry of the NP, with *α* = 0 indicating the absence of such asymmetry. The maximum of *σ*(*γ*_P_) is at the angle *γ*∈[0,π], providing the orientation of the asymmetry in the sample plane.

The finite bandwidth of the filters used means that the measured *σ*_*Λ*_ is averaged over the respective wavelength ranges. Additionally, while the transmission of the filters is approximately flat over their ranges, the illumination intensity and camera sensitivity are not. To take these features into account in our model, we compute weighted averages of simulated cross-sections over each *Λ*, as10
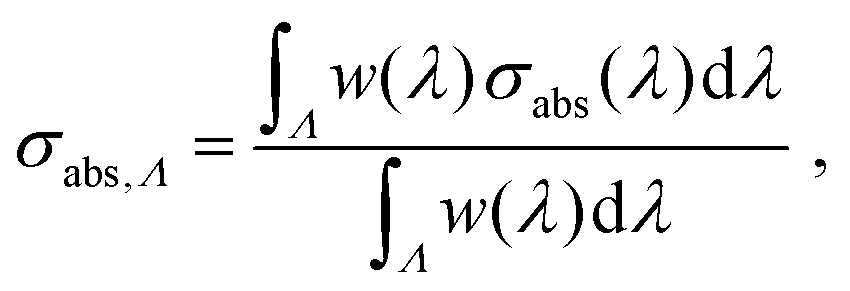
and equivalently for *σ*_sca,*Λ*_, where *w*(*λ*) = *l*(*λ*)*c*(*λ*) is the weighting incorporating the normalized illumination spectral intensity, *l*(*λ*), and camera sensitivity, *c*(*λ*), as functions of wavelength *λ*. The lamp spectrum is modeled as a blackbody radiation at a temperature of *T* = 2800 K, as described in section 2.1.4 of ref. 56. The camera sensitivity was provided by the manufacturer and was digitized for this work. The wavelength dependence of *w*(*λ*) is shown in Fig. S1.

## IV. Results and discussion

This work demonstrates the enhanced capabilities of the new ON including radial polarisation in determining individual NP morphometries in 3D. We maintain the naming and definitions of variables and quantities used in our previous publications,^43,44^ and introduce them in the text where needed. All analysis assumes that the refractive index of the NP environment in the samples is *n*_m_ = 1.52. The effect of variation in this index is discussed in section S6. For ease of reading, a list of quantities and parameters introduced in this work is provided in Table S2, and the analysis flow is represented in Fig. S13.

### A. Statistical distributions of *σ*_*Λ*_ and *α*_*Λ*_

We first introduce relevant quantities used in the statistical description of the optical measurements. For each NP, eqn (9) is fitted to the *σ*_*Λ*_(*γ*_P_) measured for linear polarisation, determining its parameters *σ*, *α*, and *γ*. Their standard errors 
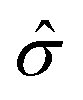
, 
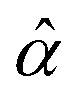
, and 
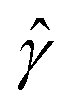
 are determined using a Monte Carlo simulation where we add random Gaussian noise to the measured data, matching the experimentally determined noise, as detailed in ref. 43 and section S3.

Given a set of *N* NPs, numbered by the index, *i*, we identify the fit parameters using the subscript *Λ*,*i*, and define the sample-wide mean of *σ*_*Λ*,*i*_ to be 
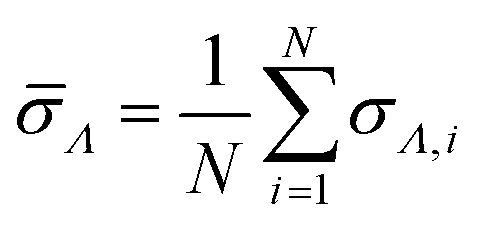
, and the associated standard deviation 
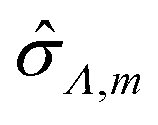
. Equivalent quantities are defined for the other two parameters. Correcting for the measurement noise, the standard deviation 
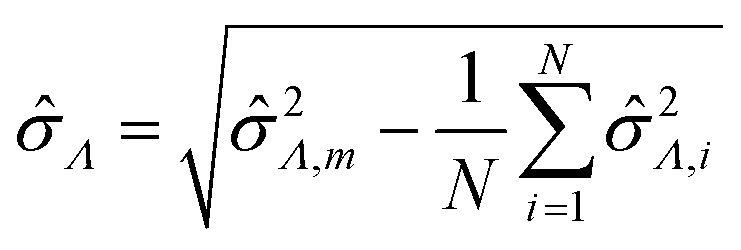
 represents the distribution of NP cross-sections originating from their sizes and shapes only.

The UGNS are highly uniform in size and shape as previously measured by HAADF–STEM.^44^ Specifically, we considered the projected diameter *D*_p_ and the PAR using the shorter, *b*′, to longer, *a*′, semi-axes observed in plane-view STEM, and found distributions characterised by mean and standard deviation values of 
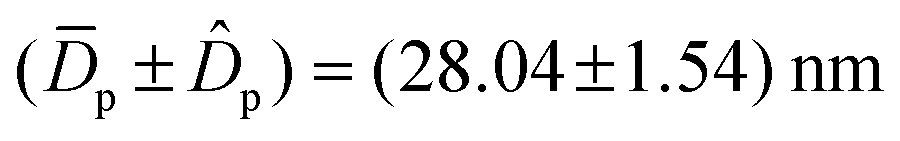
 for the diameter and 0.977 ± 0.024 for the PAR. The median *M*{.} over the *N* NPs analyzed is found to be *M*{*D*_p_} = 27.75 nm and *M*{PAR} = 0.985. From this prior knowledge, we can expect for our optical measurements: (1) a narrow distribution of both *σ*_*Λ*_ and *α*_*Λ*_, (2) a small in-plane asymmetry 
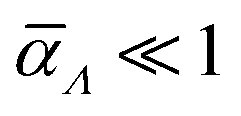
 for all *Λ* where 
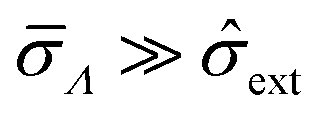
, and (3) 
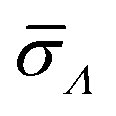
 to be close to the theoretically predicted value for a GNS of the expected size.

Fig. 2 presents *σ*_*Λ*_ and *α*_*Λ*_ for measurements of the *N* = 51 UGNS with nominal *D* = 30 nm for all *Λ* of the experiment. To mitigate small differences in the focus between the different fields of view, *σ*_ext_ was determined using the larger radius *r*_h_ (see section S4 for more details). One must correct *σ*_ext_ obtained with a finite *r*_i_ to compensate for the signal outside of the integration area. The correction factor to *σ*_ext_ was determined to be *c*_l_ = 1.12, *c*_s_ = 1.06, and *c*_h_ = 1.02, for *r*_l_, *r*_s_, and *r*_h_, respectively. In Fig. 2 we show *σ*_ext_ as measured and the modelled extinction divided by *c*_h_. Narrow distributions of *σ*_*Λ*_ are observed for all *Λ*. Small in-plane asymmetries *σ*_*Λ*_ < 0.2 are found for about 95% of the NPs at *Λ* = 600 and below, and for about 80% of NPs at *Λ* = 650. At *Λ* = 700 instead, *σ*_ext_ is only a few times larger than the noise, which results in a wide distribution of *α*_*Λ*_ estimated by the shaded areas. Hence, the sample exhibits a low optical asymmetry, with some 10% outliers.

Looking at the simulations in the inset of Fig. 2, we note that *Λ* = 550 covers the higher wavelength side of the LSPR of a spherical gold NP, suggesting that accurately modelling the width of the channels will be relevant for morphology retrieval. With increasing NP asymmetry, the LSPR polarised along the longer axis (the longitudinal mode) red-shifts, increasing its overlap with the 550 channel, and thus providing a sensitive probe of asymmetries. We find 
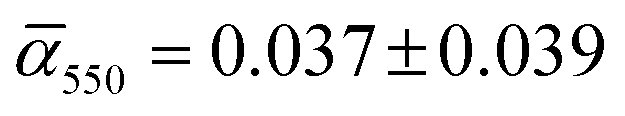
, with some 80% of NPs showing *α*_550_ < 0.054, and the rest clearly asymmetric with *α*_550_ up to 0.194. For asymmetric NPs, the LSPR will move towards the red channels (see simulations in Fig. 2 in ref. 43), increasing their *σ*_*Λ*_ and *α*_*Λ*_. Looking at the TEM image, some 10–20% of significantly non-spherical NPs can be expected. From TEM measurements, the mean AR is 0.9772 and the standard deviation is 0.0244. We thus identify any particle with an AR below one standard deviation less than the mean, *i.e.* below 0.9528, to be a non-spherical particle, and find 7 out of 61 particles, about 12%, to be non-spherical. Their red-shift can be significant, contributing to the values of *α*_650_ and *α*_700_ well above the noise. We also note that the *Λ* = 450 and 500 channels are rather insensitive to asymmetry, due to the dominant absorptive part of the permittivity, 
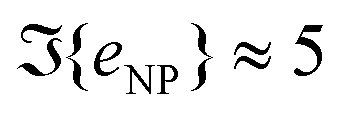
, with 
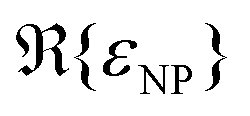
 similar to *ε*_m_, suppressing shape-dependent effects, so that *σ*_450_ and *σ*_500_ are good reporters of the NP volume.

Let us now discuss *σ*_ext_ measured using the radial polariser, *σ*_*Λ*_(*γ*_R_) (see vertical bars in Fig. 2), which we compare to the fitted *σ*_*Λ*_ for linear polarisation shown as crosses. We recall that the electric field at the sample has both in-plane and out-of-plane components, and we consider^35,54^ the relative strengths of the components along the *x*, *y*, and *z* axes, respectively as *E* = [*E*_*x*_, *E*_*y*_, *E*_*z*_]. The intensity in the back focal plane (BFP) of the condenser varies spatially, with a maximum at the center, determined by the lamp, diffuser and lenses in the illumination path, as characterised in our previous work.^35^ Using the known spatial dependence, we can calculate the intensity components at the sample polarised along the *x*, *y*, and *z* axes, normalized to have unity sum. For a linear polariser along *x* in the condenser BFP, we find *I*_lin_ = [0.826, 0.007, 0.167], corresponding to field components *E*_lin_ = [0.909, 0.084, 0.409] given by the square root of the intensity components. For radially polarised input to the BFP, we have *I*_rad_ = [0.333, 0.333, 0.334] corresponding to *E*_rad_ = [0.577, 0.577, 0.578], showing that the *z* component is increased in the radially polarised case.

For a spherical NP, the polarisation direction should not affect the measured *σ*_ext_, and thus we expect the difference *δ*_*σ*_ = *σ*_*Λ*_(*γ*_R_) − *σ*_*Λ*_ to be negligible. The histograms of *δ*_*σ*_ are shown in Fig. 2b, and we find for channels with large cross-sections *Λ* = 450, 500, and 550 a shift towards *δ*_*σ*_ > 0 of relative magnitude *δ*_*σ*_/*σ*_*Λ*_ below 3%, while for the other channels *δ*_*σ*_ is dominated by noise, shown as gray bars. To explain this small shift, we recall the “long shadow” effect,^57^ which arises from the illumination covering an oblique angular range while referencing to the intensity transmitted through the imaged sample plane. The NA of the condenser and the intensity distribution in its BFP provide the weighting of the illumination angles at the sample, from which we calculated the long-shadow factor to be 1.2428; see Fig. 6(b) in ref. 57. However, the transmission of the condenser and objective for oblique angles is polarisation-dependent, due to the reflections on the lens surfaces, which are generally lower for p polarisation compared to s polarisation. Radial polarisation is purely p polarised, while linear polarisation contains equal parts of s and p polarised light. Considering that the long-shadow factor increases with the incidence angle,^57^ the higher transmission at higher angles for radial polarisation creates an illumination with a higher long-shadow factor. This systematic error can be approximately compensated by reducing the measured *σ*_*Λ*_(*γ*_R_) by a factor 0.97, having an effect explored below. One could also speculate that a preferential orientation of the NPs on the surface creates the difference. However, we would expect in such a case that NPs preferentially lie flat on the surface, providing a larger binding surface, which would lead to a higher cross-section for in-plane fields, opposite to what is observed.

### B. Morphometric analysis

We introduced the morphometric analysis method, *i.e.* solving the inverse problem, in ref. 43. We summarize the key points here. *σ*_sca_ and *σ*_abs_ are calculated over a multidimensional space with parameters *b*/*a*, *c*/*a*, *ϕ*, *θ*, and *ψ*, where the illumination intensity spectra of the color channels *Λ* and the selected permittivity are taken into account. We then generate interpolants for *σ*_sca_ and *σ*_abs_ using the calculated grid. We compare the measured values of a given particle *i* to the calculated values for each point on the grid using the normalized error *S* defined by11
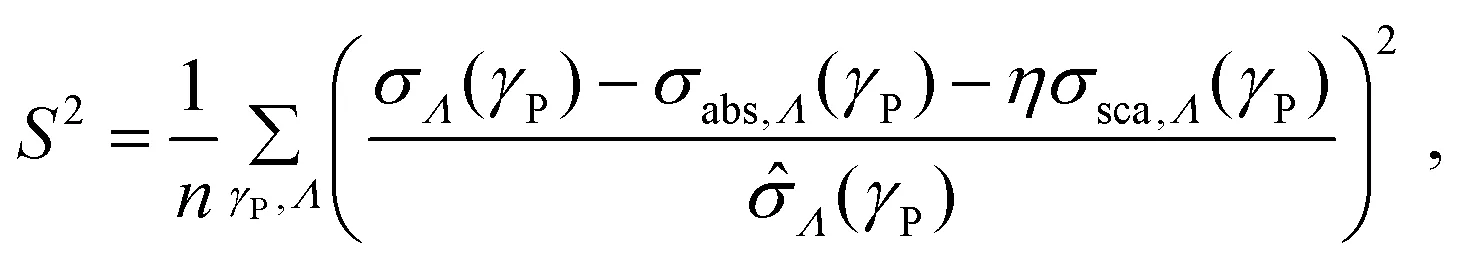
where we dropped the index *i* for brevity. *n* is the number of measurements in the sum, which includes the radially polarised measurement *γ*_P_ = *γ*_R_, and 
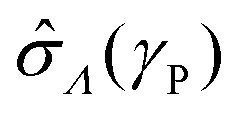
 is the measurement noise associated with *σ*_*Λ*_(*γ*_P_). The factor *η* is the fraction of scattered light which is not collected by the objective and thus contributes to the measured extinction. For our experimental configuration we calculated^56^*η* = 0.646 from the objective NA and the medium refractive index assuming dipolar scattering. We note that for the investigated particles the scattering cross-section is only a small % of the absorption cross-section, so that the exact value of *η* is not significant for the results.

Since the right side of eqn (11) is a fourth-order polynomial in *V* we can minimise *S*^2^ with respect to *V* analytically to obtain *S* and *V* for the specific dataset and obtain the volume-equivalent diameter *D*_V_. Points on the grid where *S*^2^ is smaller than a suitable cut-off are then used as initial guesses for a gradient descent employing the *σ*_sca_ and *σ*_abs_ interpolants, each providing a solution. We use the solution with the minimum *S*^2^ as a descriptor of the morphometry and orientation of the NP. To constrain retrieved morphometries, we had previously applied additional penalties using prior knowledge, *e.g.* ensemble specifications from the NP manufacturer. We do not implement this here to avoid the influence of systematic errors in such specifications.

The six spectral channels used improve the interrogation of the LSPR spectral shape compared to those in ref. 43. Hence, we re-examine here the best choice of the permittivity *ε*_NP_, defined by the minimum of12
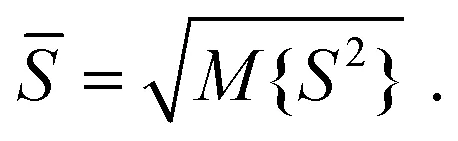


Since *S* is normalized by 
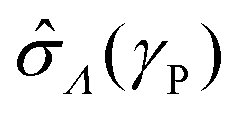
 in eqn (11), we would expect *S* = 1 for the correct model, without taking into account the number of fit parameters and the number of data points to be fitted per NP. Considering that we use *p* = 6 free parameters to fit the *n* = 30 measurements (four linear and one radial polarisation at 6 wavelengths), this expectation is reduced by the number of degrees of freedom *n* − *p* to 
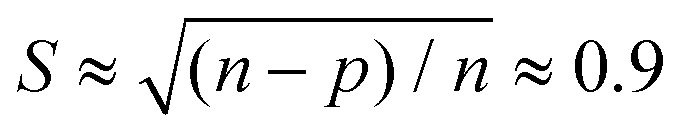
.

*S* is shown for the data analysis as discussed to this point with the solid curves in Fig. 3a as a function of the surface damping parameter *g* for each of the experimental *ε* datasets. We find minimum values of *S̄* = 1.29 at *g* = 1.8 for the MP dataset, 
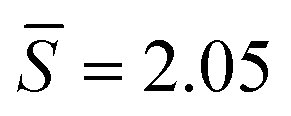
 at *g* = 1.8 for the OL dataset, and *S̄* = 1.72 at *g* = 0.3 for the JC dataset. Compared to our results in ref. 44, *S̄* is more sensitive to *g*, due to the lower noise and larger number of measurements used. The values of *S̄* between 1.3 and 2, above the expected value of 0.9, indicates systematic errors of one to two times the noise in the measurements, which limit the accuracy of size and shape retrieval. The histograms of *S* are shown in Fig. 3 for each dataset and *g* at the minimum of *S̄*(*g*) and either side of it. At the minimum, a nearly Gaussian shape is observed. The distributions of *S* for each dataset display a few outliers of large *S*, indicating that some NPs are not well described by the model.

**Fig. 3:**
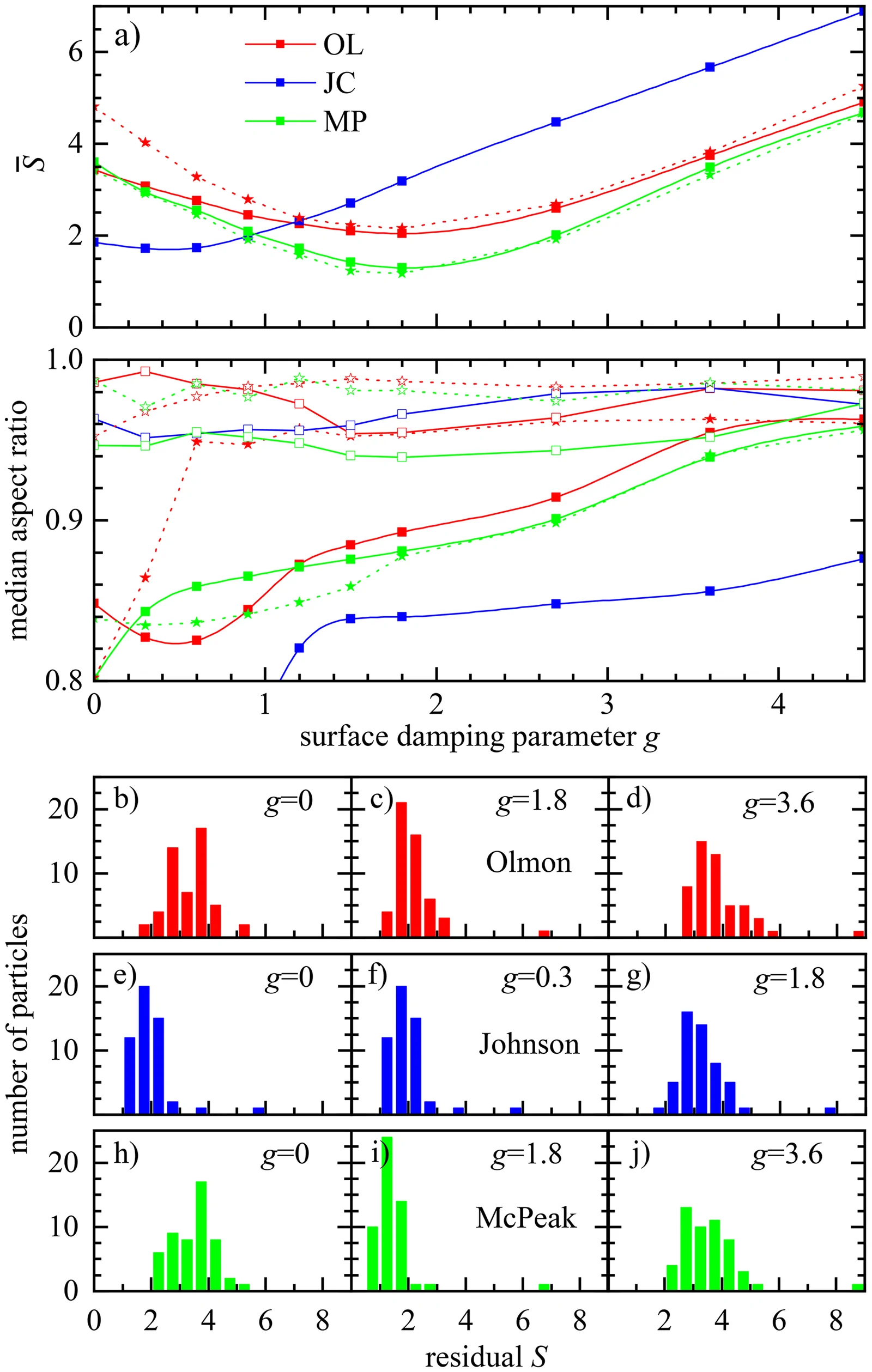
*S̄* and selected statistics of *S* for *N* = 51 UGNS of nominal *D* = 30 nm for the varying surface damping parameter *g* and different *ε* datasets as indicated by color. (a) Top: *S̄ versus g*. Solid lines and squares for *ε*_NP_(*λ*). Dotted lines and stars for *ε*^s^_NP_(*λ*) with 0.97× correction for *σ*_ext_(*γ*_R_) data. Bottom: median of the fitted average aspect ratio, for MAR (filled symbols) and PAR (empty symbols). Solid and dotted line designations same as at the top. (b–j) Histograms of *S*, with (b–d) using the OL dataset with *g* = {0,1.8,3.6}, (e–g) JC dataset with *g* = {0,0.3,1.8}, and (h–j) MP dataset with *g* = {0,1.8,3.6}. The values of *g* shown are at *g* = 0, *g* at the minimum of *S̄*(*g*) and a larger *g*.

Notably, the smallest *S̄* is found for MP, which indicates the best choice since it represents the model which best fits the data. On the other hand, we see in Fig. 3, S7, and S8 that OL retrieves the roundest shapes as shown by *M*{MAR}, closest to the shape measured by TEM. Excluding the radial polarisation data yields the lowest *S̄*, for all permittivities (see Fig. S7). This can be rationalised noting that the radial polarisation probes the shape in the out-of-plane direction, restraining the fit from using this shape to create spectral shifts while the in-plane shape is constrained by *σ*_*Λ*_(*γ*_P_).

In principle, all permittivity datasets should be consistent apart from surface damping, as they have all been measured on gold films of different crystallinity. While this is the case for OL and JC, with JC corresponding to OL with *g* ≈ 1, reflecting the lower crystallinity of the film measured in JC compared to the single crystal data in OL, MP corresponds to a higher plasma frequency, leading to a blueshift of the LSPR of a spherical NP by about^44^ 4 nm. Using MP in the fit thus yields higher NP asymmetry, which compensates the blueshift by a redshift due to the non-spherical shape.

The results of the morphometric fits for the *N* = 51 UGNS with nominal *D* = 30 nm are shown in panels (a–b) of Fig. 4. The retrieved NP shapes for OL *g* = 1.8, shown as filled circles, are slightly non-spherical, with *c*/*b* = 0.91 ± 0.032, *c*/*a* = 0.81 ± 0.12, and *b*/*a* = 0.96 ± 0.04, where the errors are standard deviations of the ensemble, and a mean (median) MAR of 0.89 (0.90). When excluding *σ*_*Λ*_(*γ*_R_), the results show slightly rounder NP shapes, with *c*/*b* = 0.88 ± 0.07, *c*/*a* = 0.85 ± 0.07, *b*/*a* = 0.97 ± 0.03, and a mean (median) MAR of 0.91 (0.91). Here, the shapes form two clusters, with the smaller cluster having a *c*/*b* lower than any shapes retrieved when including *σ*_*Λ*_(*γ*_R_). We find *D*_V_ = (25.6 ± 0.5) nm (median: 25.6 nm) over the ensemble when including *σ*_*Λ*_(*γ*_R_), showing a systematic deviation of about 2 nm, or 8%, from the TEM results. The results for the JC and MP datasets generally show retrieved shapes which are more widely distributed and of stronger non-sphericity compared to OL, and MP retrieves nearly larger average diameters. We explore in section S5 the effect of the different corrections and selections introduced on the shape retrieval in more detail.

**Fig. 4:**
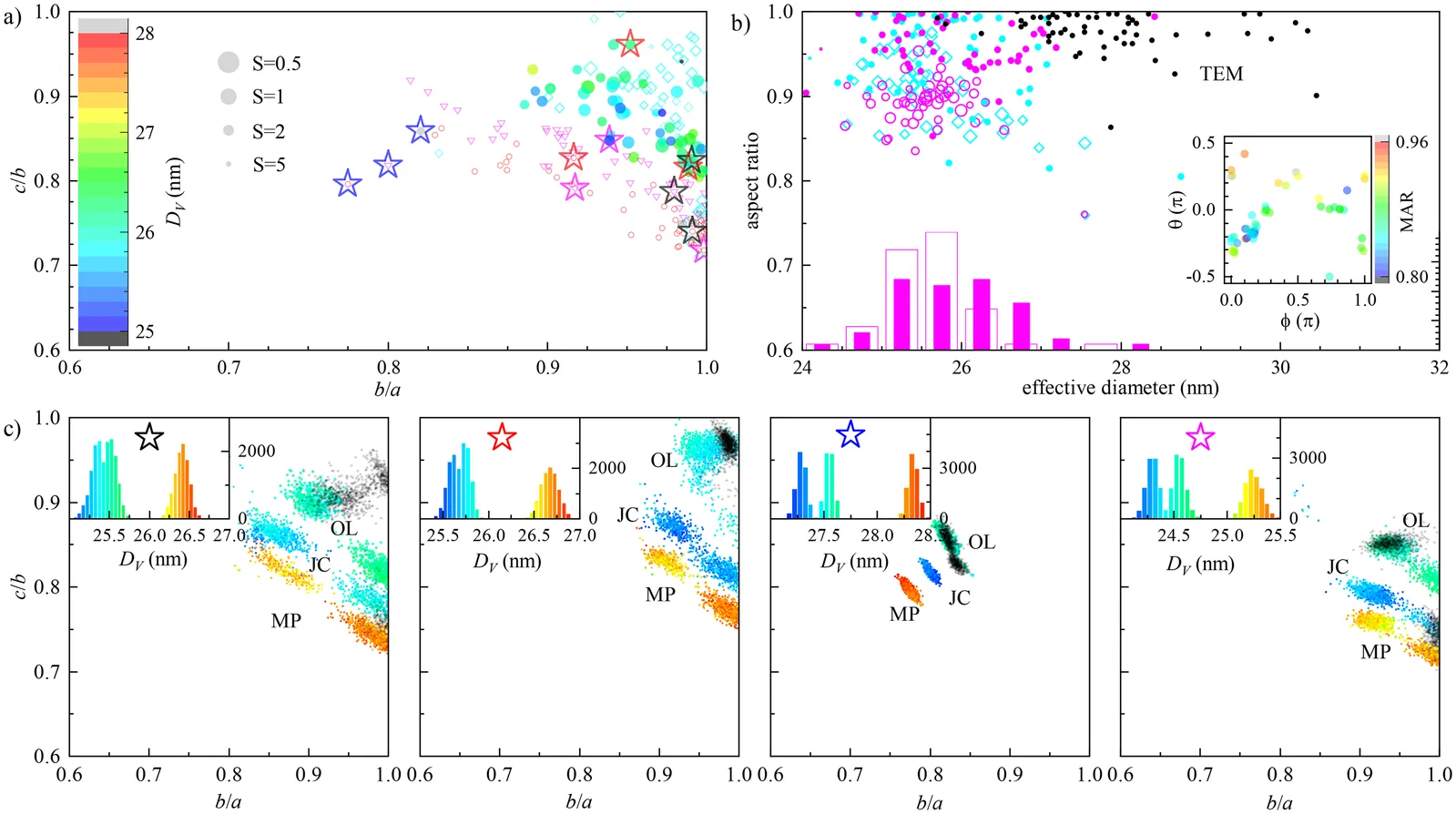
Morphometric results for *N* = 51 UGNS with nominal *D* = 30 nm. (a) Retrieved ARs for OL *g* = 1.8 are shown as filled symbols with a colour indicating the fitted diameter *D*_V_. ARs retrieved using only *σ*_*Λ*_(*γ*_P_) are shown as cyan empty diamonds. Also shown are retrieved ARs for JC *g* = 0.3 (magenta empty triangles), and for MP *g* = 1.8 (red empty circles). (b) MAR *versus D*_V_ for OL *g* = 1.8 (magenta empty circles), with the histogram showing the number distribution of *D*_V_ (empty) and *D*_p_ (filled). Corresponding PARs are shown as filled symbols *versus D*_p_. Results using only *σ*_*Λ*_(*γ*_P_) are shown as diamonds. The black filled squares indicate TEM measurements of the PAR *versus D*_p_. Inset: pitch (*θ*) and roll (*ϕ*) angles showing the particle orientation for OL, with a symbol colour encoding the MAR. The size of the circles in (a) and the empty symbols in (b) is given by *S̄* = 1/(1 + *S*), as indicated in (a), with larger size indicating a lower fit error *S*. (c) Simulated effect of the measurement noise on the retrieved ARs for individual NPs indicated by the colored stars in (a). Results for 1000 realisations of Gaussian noise with 
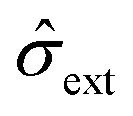
 standard deviation added to the measured extinction data are shown. Inset: *D*_V_ histograms, providing a colour scale for the symbols. Results for OL, JC, and MP at *g* of 1.8, 0.3, and 1.8, respectively, with corresponding results labelled. Black symbols are results using only *σ*_*Λ*_(*γ*_P_) for OL.

Notably, the projected shapes are generally rounder and of larger diameters; see filled circles (PAR) and filled histogram (*D*_p_) in Fig. 4b. This can be interpreted in two ways: (i) since the in-plane asymmetry is measured with higher precision *via* the *γ*_P_ dependence, the PAR has less error and thus reflects the correct aspect ratio in all three dimensions of the NP and (ii) the NPs are oriented on the surface in a way appearing more round in-plane. Considering the nearly spherical shapes, a physical mechanism which would result in condition (ii) both for TEM and optical measurements is not obvious.

In Fig. 4c, we examine the uncertainty in the retrieved ARs and *D*_V_ due to measurement noise for four selected NPs, indicated by the colored stars in Fig. 4a. The analysis was performed for all three permittivity datasets with *g* minimizing *S̄*. We simulate the effect of the measurement noise by adding a realisation of Gaussian random noise with a standard deviation of 
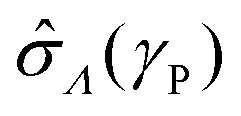
 (see the inset of Fig. 2a) from the measured *σ*_*Λ*_(*γ*_P_), and then repeat the morphometric analysis. Each set of scatter data in each of the four panels represents 1000 noise realizations. The ARs often exhibit bimodal distributions, with the greatest separation between modes apparent for MP and JC, while the width of the individual modes is typically below 5%. Notably, the modes are often close to *b* = *a* or *c* = *b*, allowing the achievement of zero in-plane asymmetry (which is defined with high precision by the linear polarisation data) for appropriate NP orientations. Using synthetic *σ*_ext_ data generated using the Rayleigh–Gans model and the specified experimental corrections, such multimodal results are not observed,^43^ suggesting that they are caused by systematic errors. Notably, the sample distributions in Fig. 4a for each permittivity mirror the spread of the noise distributions for the rounder NPs in Fig. 4c for each permittivity, which points to a homogeneity of the sample, as well as this systematic effect. We will return to this topic again later in the text. Generally, for near-spherical NPs, AR retrieval is less precise due to the quadratic parameter dependencies close to symmetry points of the multi-dimensional parameter space.^43^ Significantly asymmetric NPs instead yield more precise retrieval, as exemplified by the blue-starred NP. In this case, precision is better than 3%, corresponding to 0.84 nm length along the NP axes. For further insights into this effect, see section S3. Variations in *n*_m_ result in systematic changes in *c*/*b* of about twice the index change, as discussed in section S6. For a realistic refractive index accuracy of 0.01, the corresponding *c*/*b* systematic error is 2%. Remarkably, the retrieved *D*_V_ shows a precision of (0.04–0.1) nm standard deviation for the selected NPs.

The systematic deviation of about 2 nm between the mean of the retrieved *D*_V_ and that found by TEM might be due to several factors. A known systematic error is the quasi-static approximation used in the model. Considering the wavelength of the LSPR for a spherical gold NP of about *λ*_0_ = 530 nm, the wavelength in the medium of *n*_m_ = 1.52 is *λ*_0_/*n*_m_ ≈ 350 nm, which is about 12 times the nominal diameter of the NPs. This suggests that taking into account retardation, which leads to radiative damping and red-shift of the LSPR, might be relevant to achieve accuracies below 10% for the investigated NPs. Indeed, the extinction spectrum of a gold NP with the TEM-measured average diameter *D*_V_ = 27.94 nm, calculated including retardation using Mie theory (see the inset of Fig. 2a, dotted black line), shows about a 4% increase and a 5 nm red-shift of the LSPR peak; see also section S7. The vertical lines in Fig. 2a show the corresponding weighted cross-sections (solid for Rayleigh–Gans, dotted for Mie) for all colour channels. We would expect that the redshift would result in rounder retrieved NPs, while the increase would result in about 1% smaller *D*_V_ considering the approximate scaling of the cross-section with the volume.

To investigate this further while keeping the Rayleigh–Gans model, we approximate the effects of retardation by spectrally shifting the permittivity with respect to *λ* to obtain *ε*^s^_NP_(*λ*) = *ε*_NP_(*λ* + *δλ*), with *δλ* = −5 nm, and repeating the ON analysis using *ε*^s^_NP_ and the 0.97× correction for *σ*_ext_(*γ*_R_). The resulting dependence of *S̄* and the median MAR on *g* is given as dotted lines in Fig. 3a for OL and MP, showing that the *g* at which *S̄* reaches a minimum is rather unaffected, while the median MAR increases. The corresponding ON results are given in Fig. 5 for OL and MP. The retrieved shapes are rounder for both datasets, with OL showing clearly round shape retrieval with a median MAR of 0.954, compared to 0.892 for MP. Meanwhile, the median diameter for MP is *M*{*D*_V_} = 26.72 nm, larger than 25.77 nm for OL. For OL, nearly all of the asymmetry lies in *b*/*a* corresponding to a prolate particle, and most orientations show *θ* ≈ −π/2, corresponding to a prolate NP standing at the end, *i.e.* its long axis is normal to the surface. This suggests a systematic effect, where a negligible in-plane polarisation-dependence combines with a constraint by *σ*_ext_(*γ*_R_). MP by comparison shows a less constrained distribution of orientations, with widely distributed tilts *θ*.

**Fig. 5:**
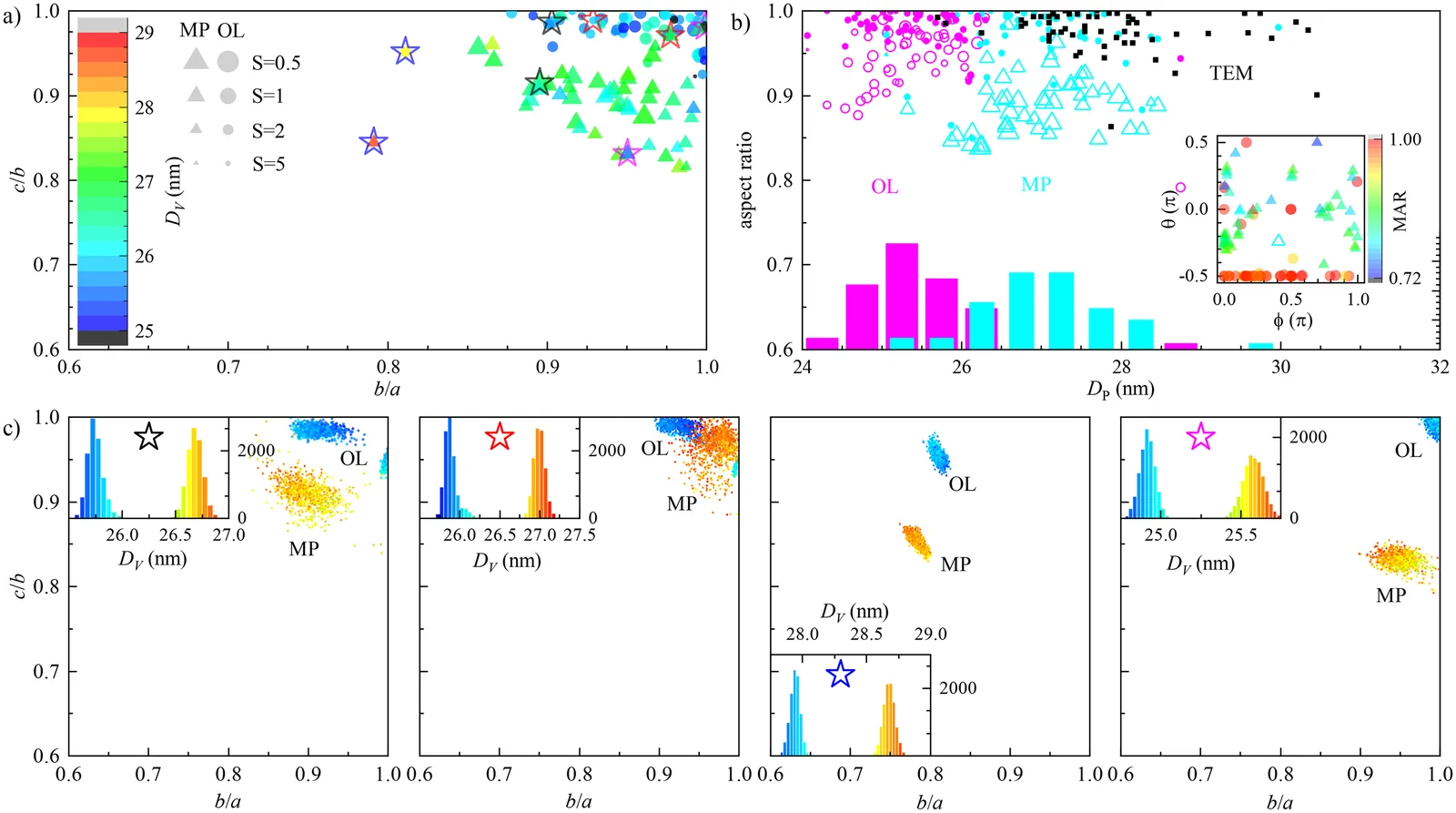
Similar to Fig. 4, but for OL and MP using *ε*^s^_NP_(*λ*) = *ε*_NP_(*λ* + *δλ*) with *δλ* = −5 nm and the 0.97× correction for *σ*_ext_(*γ*_R_). (a) Retrieved ARs shown as filled symbols (MP triangles, OL circles) with a color indicating the fitted diameter *D*_V_. (b) MAR (empty symbols) and PAR (filled symbols) *versus D*_p_, with the histogram showing the number distribution of *D*_p_, with OL shown as magenta circles/bars and MP as cyan triangles/bars. The black filled squares indicate TEM measurements of the PAR *versus D*_p_. Inset: pitch (*θ*) and roll (*ϕ*) angles showing the particle orientation. (c) Simulated effect of the measurement noise on the retrieved ARs for individual NPs indicated by the colored stars in (a). Results for 1000 realisations of Gaussian noise with 
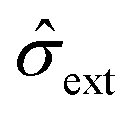
 standard deviation added to the measured extinction data are shown. Inset: *D*_V_ histograms, providing a colour scale for the symbols.

The projected aspect ratios (PARs) versus the corresponding diameters *D*_p_ are also shown in Fig. 5b. The median PAR values are *M*{PAR} = 0.987 for OL and 0.981 for MP, with their dependence on *g* given in Fig. 3a. These values are significantly higher than those of MAR. The histograms of *D*_p_ are shown and the median for MP is *M*{*D*_p_} = 27.02 nm, larger than 25.37 nm for OL. Remarkably, both the PAR and *D*_p_ for MP are in good agreement with the TEM results (*M*{PAR} = 0.985, *M*{*D*_p_} = 27.75 nm).

Furthermore, for the two rounder particles in Fig. 5c, OL shows distinctly bimodal distributions, while MP returns to unimodal distributions. This suggests that the origin of the bimodal distributions in Fig. 4d is a systematic error in the permittivity used in Fig. 4c. For these examples, at one standard deviation from the mean, the precision in *D*_V_ is improved to (0.04–0.07) nm, and the precision in AR is (0.37%–2.8%) for OL and (0.45%–2%) for MP, depending on the NP.

## V. Conclusions

We have presented an enhanced ON method with optical cross-sections measured using six spectral channels, and both linear and radial polarisation to probe both in-plane and out-of-plane NP responses. For nominal 30 nm ultra-uniform gold nanospheres, we demonstrated significant improvement in the precision and accuracy of the method. Precision in the size and shape has been improved to below 0.1 nm and 5%, respectively. We examined the effect of the permittivity dataset on the morphometry retrieval and found that McPeak *et al.*^46^ with a surface damping factor *g* = 1.8 yields the best results. We found that the small effect of retardation for these NPs, which are some 12 times smaller than the light wavelength in the surrounding medium, provides a LSPR red-shift of some 5 nm. This is significant due to the high precision of the measurements, and correcting for it improves accuracy to within 5% in shape and size using TEM measurements as a reference. We, however, note that this accuracy is contingent on the permittivity used to describe the material response.

The retardation effects, which are small for the investigated 30 nm GNPs, increase with size, so that below 20 nm size they can be neglected, but above 50 nm size they require a full numerical treatment for accurate results. Metal NPs generally have LSPRs which are highly shape-dependent, so that the spectral dependence of the cross-sections provides a sensitive shape retrieval. The spectral width of the LSPRs increases with the imaginary part of the permittivity, providing high sensitivity for silver and gold NPs, having narrow LSPRs, and lower sensitivity for most other metals. Dielectric NPs do not have LSPRs, and the shape retrieval mostly relies on the measured polarisation dependence. For materials with refractive indices of 1.3 to 2 such as typical organic materials and glasses, the cross-sections for a given size are lower than those for metals. Therefore, the same measurement protocol generally provides a lower size and shape precision for dielectric NPs compared to those for metal NPs.

We note that the shapes retrieved here are constrained by the ellipsoid model used and thus represent effective parameters of the particle morphology. Future development could see the use of numerical models, capable of treating NPs outside of the limitations of analytical models. Another approximation limiting the accuracy is the use of a local permittivity to describe the NP optical response, which is known to break down on the sub-nm scale in general. Again, more complex theory models^58^ can be used to improve on this. The precision of the method is so high that small systematic effects in the experimental setup, such as the angular resolved transmission and the angular intensity distributions, are relevant to achieve an accuracy comparable to, or better than, the precision. These can be corrected by further characterisation of the instrumentation.

Overall, this study shows the ability of the ON to complement TEM, and as a methodology for high-throughput morphometric studies of NPs within a known shape and material class. It also offers an interesting approach to determine the permittivity of materials on the nanoscale, and to uncover non-local effects.

## Author contributions

L.P., W.L. and P.B. conceived the work. F.A. prepared the samples and performed the optical measurements. L.P, P.B. and W.L. developed the numerical model and fitting methods. L.P. performed the numerical simulations and data fitting. All authors contributed to the data interpretation and writing of the article.

## Conflicts of interest

There are no conflicts to declare.

## Supplementary Material

NR-018-D6NR03059A-s001

## Data Availability

Information on the data underpinning the results presented here, including how to access them, can be found in the Cardiff University data catalogue at https://doi.org/10.17035/cardiff.31304983. Supplementary information (SI) is available. Contains sections S1 to S7, figures S1 to S13, and tables S1 to S2. See DOI: https://doi.org/10.1039/d6nr03059a.
