# Supplementary material for: Sub-nanometer 3D morphometric precision of polarisation-resolved wide-field optical extinction microscopy determines the roundness of individual gold nanospheres
Source: Nanoscale. 2026 Sep 15;18(40):21956–68. doi: 10.1039/d6nr03059a (PMC13635932; doi:10.1039/d6nr03059a)
Supplement: Supplementary file 1 [file NR-018-D6NR03059A-s001.pdf]

Supplementary Information (SI) for Nanoscale.
This journal is © The Royal Society of Chemistry 2026

Sub-nanometer 3D morphometric precision of polarisation-resolved wide-field optical extinction microscopy determines the roundness of individual gold nanospheres - Supporting information

Lukas M Payne,[1,2] Furqan Alabdullah,[3,2,4] Paola Borri,[2] and Wolfgang Langbein[1,5]
[1)] *Cardiff University School of Physics and Astronomy, The Parade, Cardiff CF24 3AA, United Kingdom*
[2)] *School of Biosciences, Cardiff University, Museum Avenue, Cardiff CF10 3AX, United Kingdom*
[3)] *School of Engineering, Cardiff University, The Parade, Cardiff CF24 3AA, United Kingdom*
[4)] *Engineering Technical College of Al-Najaf, Al-Furat Al-Awsat Technical University, Najaf 31001, Iraq*
[5)] *Corresponding author, langbeinww@cardiff.ac.uk*

(Dated: 8 September 2026)

S1. ILLUMINATION SPECTRUM AND CAMERA SENSITIVITY

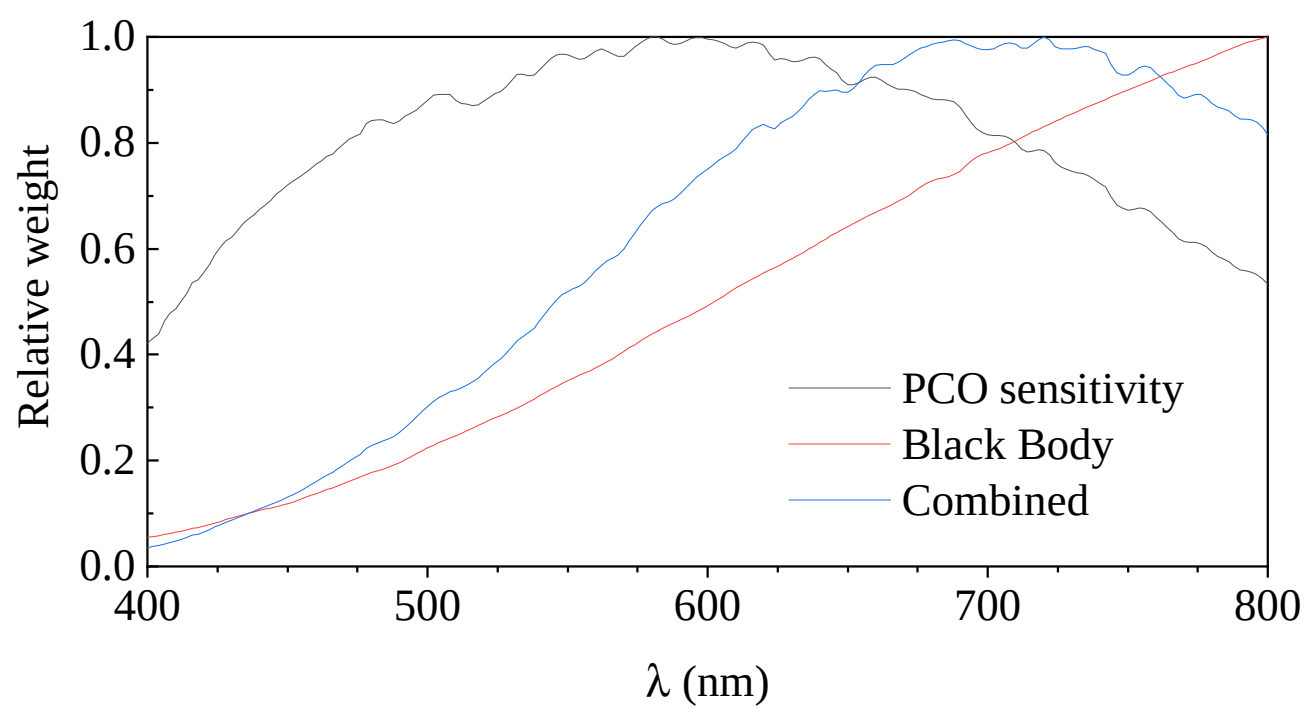

FIG. S1. Plot of $l(\lambda)$ (red curve), $c(\lambda)$ (black curve), and $w(\lambda)$ (blue curve).

The illumination setup in this experiment uses a halogen lamp as source, operated at close to max setting (100 W), with 40 nm bandwidth filters to provide coarse wavelength selection in the experiment. While the filters exhibit close to flat transmission across their bandwidth, the lamp spectrum is varying over the filter ranges, specifically in the green-blue region. To take this into account, we approximate the lamp spectrum as a blackbody radiator as in Section 2.1.4 of Ref. 1. One must also consider the spectral properties of the detection. The PCO Edge 5.5 scientific CMOS (sCMOS) exhibits a quantum efficiency (QE) depending on wavelength, which was characterised by the manufacturer and was digitised for this work. Fig. S1 shows the calculated blackbody lamp spectrum $l(\lambda)$, the camera sensitivity $c(\lambda)$, and the combined dependence $w(\lambda) = l(\lambda)c(\lambda)$, normalized for visualisation purposes.

S2. PROJECTED ELLIPSOIDAL PARAMETERS

Given the rotation angles (ϕ, θ, ψ) and semi-axis lengths (a,b,c) of the fitted ellipsoid, as introduced in Sec. II, we can calculate its projection along the z axis in the laboratory frame, which is normal to the substrate surface, as follows.[2] We introduce the ellipsoid matrix

$$M = \begin{bmatrix} a^{-2} & 0 & 0 \\ 0 & b^{-2} & 0 \\ 0 & 0 & c^{-2} \end{bmatrix} \tag{S1}$$

describing the ellipsoid surface as points $\mathbf{r}$ respecting $\mathbf{r}\cdot M\mathbf{r} = 1$, and rotate it into the lab frame,

$$RMR^{\mathsf{T}} = \begin{bmatrix} A & \mathbf{b} \\ \mathbf{b}^T & d \end{bmatrix} \tag{S2}$$

using the rotation $R = R_\psi R_\theta R_\phi$ as introduced in Sec. II. The rotated matrix is expressed by an in-plane matrix A, a vector $\mathbf{b}$, and a scalar d as shown. We proceed by introducing the projected ellipse matrix

$$Q = A - \frac{\mathbf{b}\mathbf{b}^T}{d} \tag{S3}$$

which we diagonalise to determine its two eigenvalues $\lambda_1 < \lambda_2$, which define the in-plane semi-axis lengths $a' = 1/\sqrt{\lambda_1}$ and $b' = 1/\sqrt{\lambda_2}$.

S3. NOISE OF LINEAR POLARISATION FIT PARAMETERS

The extinction cross-sections, σ_{ext}, measured for different linear polariser angles, γ_{P}, are fitted by Eq. 9 to retrieve the in-plane dipolar asymmetry, α, its orientation, γ, and the average cross-section, σ.

To determine how the experimental noise, $\hat{\sigma}_{\mathrm{ext}}$ arising from photon noise and sample background structure, affects the fit parameters, we simulate realisations of $\sigma_{\mathrm{ext}}(\gamma_{\mathrm{P}})$ for asymmetries $\alpha_{\mathrm{s}} \in [0, 0.1, 0.2, 0.3, 0.4, 0.5, 0.8, 1.0]$ and orientations $\gamma_{\mathrm{s}} \in [0, 10, 15, 20, 30, 45, 90]$ degrees. The simulations are repeated for different numbers of polarisation angles $N_{\mathrm{P}} \in [3, 4, 6]$, with $\gamma_{\mathrm{P}} \in [0, 180/N_{\mathrm{P}}, ..., 180(N_{\mathrm{P}} - 1)/N_{\mathrm{P}}]$. We generate $\sigma_{\mathrm{ext}}(\gamma_{\mathrm{P}}, \alpha_{\mathrm{s}}, \gamma_{\mathrm{s}})$ by adding Gaussian noise of standard deviation $\hat{\sigma}_{\mathrm{ext}} = 0.1\sigma$ to the results of Eq. 9, and fit the data using Eq. 9, for $N = 10000$ noise realisations.

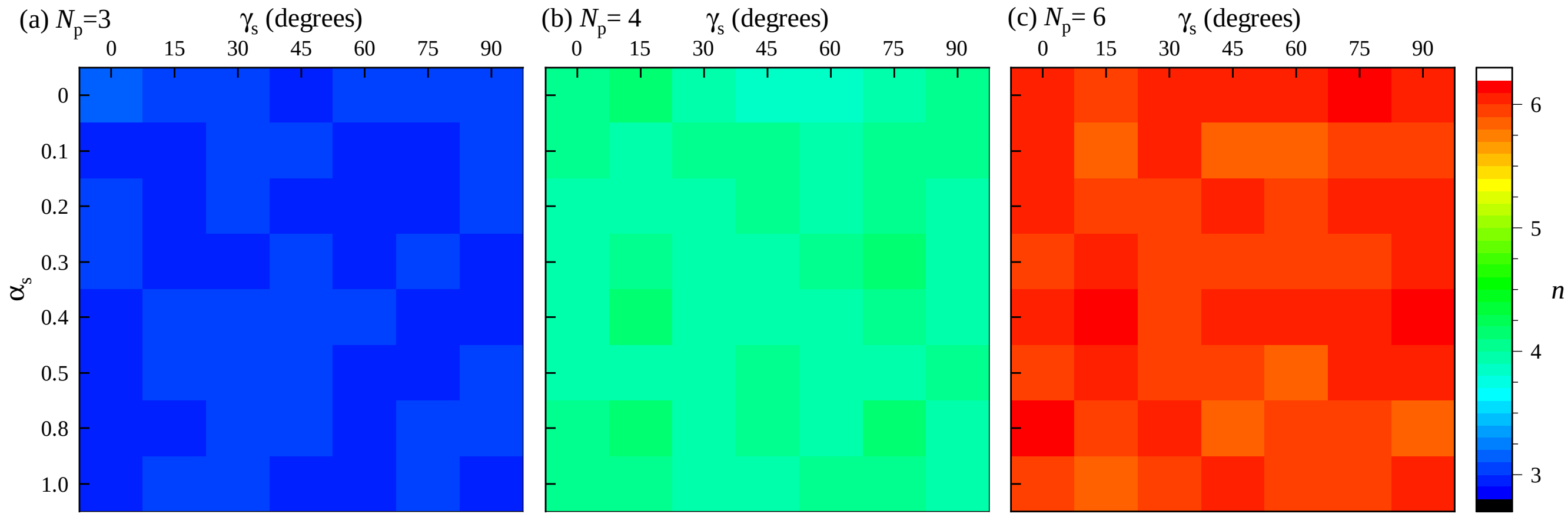

FIG. S2. Effective number of measurements $n = (\hat{\sigma}_{\rm ext}/\hat{\sigma})^2$ in the noise in σ, as function of $\alpha_{\rm s}$ and $\gamma_{\rm s}$, determined from fits to simulated extinction data using Eq. 9. Results are shown for 10% relative noise in $\sigma_{\rm ext}$, for different numbers $N_{\rm P}$ of equidistant polariser angles across 180°, with panels (a), (b), and (c) using $N_{\rm P} = 3$, 4, and 6 angles, respectively.

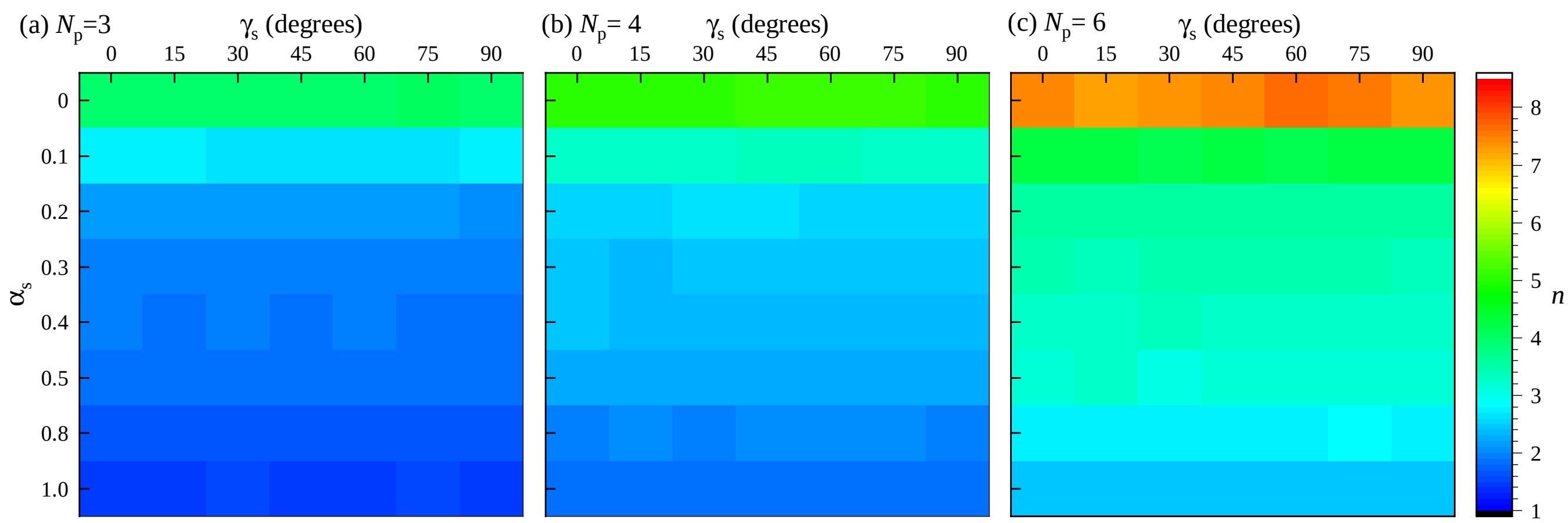

FIG. S3. As Fig. S2, but for α, using $n = (\hat{\sigma}_{\rm ext}/(\sigma\hat{\alpha}))^2$.

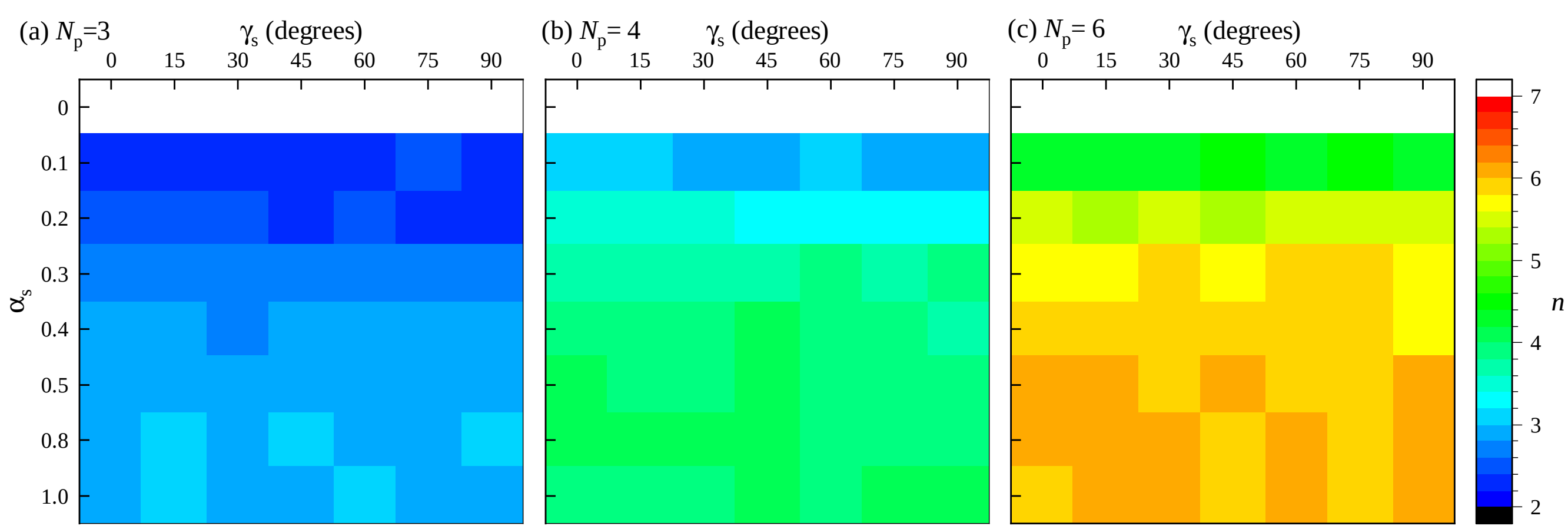

FIG. S4. As Fig. S3, but for γ, using $n = 0.5(\hat{\sigma}_{\rm ext}/(\sigma\alpha\hat{\gamma}))^2$, with γ in radians, and $\hat{\gamma}$ calculated as circular error.[3]

For small asymmetries α_s, σ is effectively the mean of $\sigma_{ext}(\gamma_P)$ over all γ_P, so that the noise in σ should be given by $\hat{\sigma}_{ext}/\sqrt{N_P}$, where N_P is the number linear polariser angles used. We accordingly define an effective number of measurements $n(\alpha_s,\gamma_s) = (\hat{\sigma}_{ext}/\hat{\sigma})^2$ for σ, and the simulation results shown in Fig. S2 confirm that n is close to N_P, notably not only for $\alpha_s = 0$, but across all α_s.

For α, the effective number of measurements is defined as $n(\alpha_s,\gamma_s) = (\hat{\sigma}_{ext}/(\sigma\hat{\alpha}))^2$, noting that the oscillation amplitude is $\sigma\alpha$. The simulation results are shown in Fig. S3. Naively, one could expect $n = N_P - 2$, considering that we measure 2 additional parameters apart from α. This is also approximately the case for α_s around 0.3-0.5. For smaller $\alpha_s < 0.3$, n increases, approximately doubling at $\alpha_s = 0$. This is attributed to the choice $\alpha \leq 0$, controlling the sign of α by a 90 degree shift of the angle γ. Therefore, the distribution is narrowed, at the expense of noise in γ. For larger $\alpha > 0.5$ instead, n decreases by about 1. We attribute this to a reduction of the noise in γ at the expense of the noise in α.

For γ, the noise $\hat{\gamma}$ is calculated in radians using a circular statistics[3] for the periodicity of 180 degrees, and the effective number of measurements is defined as $n(\alpha_s,\gamma_s) = 0.5(\hat{\sigma}_{ext}/(\sigma\alpha\hat{\gamma}))^2$. The simulation results shown in Fig. S4 reveal that n is close to N_P, except for small α, where noise in γ becomes so large that $\hat{\gamma}$ is limited by the periodicity.

Interestingly there is no obvious dependence of n on γ_s for any of the parameters, not even when measuring only $N_P = 3$ angles spaced by 60 degrees, indicating that using only 3 angles is a suitable choice.

S4. INFLUENCE OF INTEGRATION RADIUS

The integration radius, r_i, affects the measurement of σ_{ext} in wide-field extinction microscopy. For simplicity, let us first consider the more straightforward case of darkfield microscopy, where scattering by a NP results in an intensity point spread function (PSF) in the image given by a two-dimensional (2D) Airy distribution; in reality there will be aberrations arising from the specific optics and polarisation dependent effects. The envelope of this distribution decays asymptotically to zero with the distance from the central peak, so that at a finite radius some of the scattered power is not captured. One must then correct the measured integral by a factor, for example based on the known fraction of the total integral. By contrast, in extinction we measure a coherent signal, given by the interference of the scattered light with the incident light in forward direction, an effect also called the optical theorem. This interference results in a more extended PSF determined by additional factors, specifically the in-plane coherence length of the illumination given by the numerical aperture of the condenser, and the defocus. We have shown previously[4] that for suitable choice of illumination NA one can achieve both a minimised fringing and therefore minimally extended PSFs, increasing the maximum possible area density of NPs in a sample, and a finite integration radius at which the extinction is fully measured.

In this work, we chose to use the maximum opening of the condenser (NA = 1.34) to maximise the out-of-plane component of the electric field at the sample, which we employ using radially polarised light to probe the out-of-plane shape of the NPs. However, using the maximum condenser NA leads to more extended fringing[4] in the extinction PSF, which decreases the maximum area density of NPs without spatially overlapping signals, and results in a measured extinction converging more slowly with integration radius. The latter point is the more important one for this work, since the sample area density of NPs was low. We typically use an integration radius, $r_s = 3\lambda/2\text{NA}$, which collects about 94% of the extinguished power for maximum opening of the condenser. For the optimal conditions mentioned above, r_s would collect the full extinction, as the fringing at larger radii has compensating positive and negative contributions. This discussion assumes optimal focussing, which for a purely absorbing NP results in the largest peak extinction. For slight defocussing, the PSF spreads out, as the scattering changes its phase from being in-quadrature to the incident light, and thus not creating intensity changes, to being partly in or out of phase, creating bright and dark fringes (an effect which can be used to get contrast from non-absorbing scatters, popular in electron microscopy). In our data, a few fields of view (FOV) at a few Λ exhibit slightly wider PSFs (10%-20% larger radius of first PSF minimum) compared to most others, indicating slight defocus. Such defocus effects can be countered using a larger radius $r_i > r_s$ in the analysis of σ_{ext}.

TABLE S1. Interquartile range differences $(\text{IQR}\{\sigma_i\} - \text{IQR}\{\sigma_s\})/\text{nm}^2$ as a function of Λ, with subscript $i \in (\text{h,l})$, indicating which measurement radius, r_h or r_l, was used, and the IQR was calculated over all $N = 51$ NPs from all 8 FOVs.

i	450 nm	500 nm	550 nm	600 nm	650 nm	700 nm
h	-5.47	-6.99	3.69	-23.02	-10.14	-2.21
l	7.49	11.28	6.10	33.00	21.67	10.74

We compare the polarisation-averaged σ_Λ measured using r_s, denoted as σ_s, with the one measured using a larger $r_h = 1.2\,r_s$, denoted as σ_h, in Fig. S5, and the one measured using a smaller $r_l = 0.8\,r_s$, denoted as σ_l, in Fig. S6. For both figures, symbol color indicates measurements from a single FOV. The difference between the interquartile ranges (IQR) of σ_s and σ_h or σ_l is given in Table S1. For σ_h, negative values across most Λ are found, showing that using r_h yields a smaller spread of the data, compared to r_s. Conversely, for σ_l, the values are positive, showing that using r_l yields a larger spread of data compared to r_s. For channels $\Lambda \in \{450, 500\}$ nm we can see that most of the data in Fig. S5 appears as a single group, specifically in σ_h, while for $\Lambda > 500$ nm two groups of data are visible, with black, red, and green triangles form the lower group, separating further from the rest for $\Lambda = 550$ nm and $\Lambda = 650$ nm. For $\Lambda = 700$ nm noise dominates. Note, the lines of best fit, added for $\Lambda = 550$ nm, indicate that certain FOVs are grouped together. We attribute this apparent wavelength and FOV dependent discrepancy to a slight defocus of the corresponding data. As might be expected from this attribution, the effect is more pronounced for σ_l in Fig. S6,

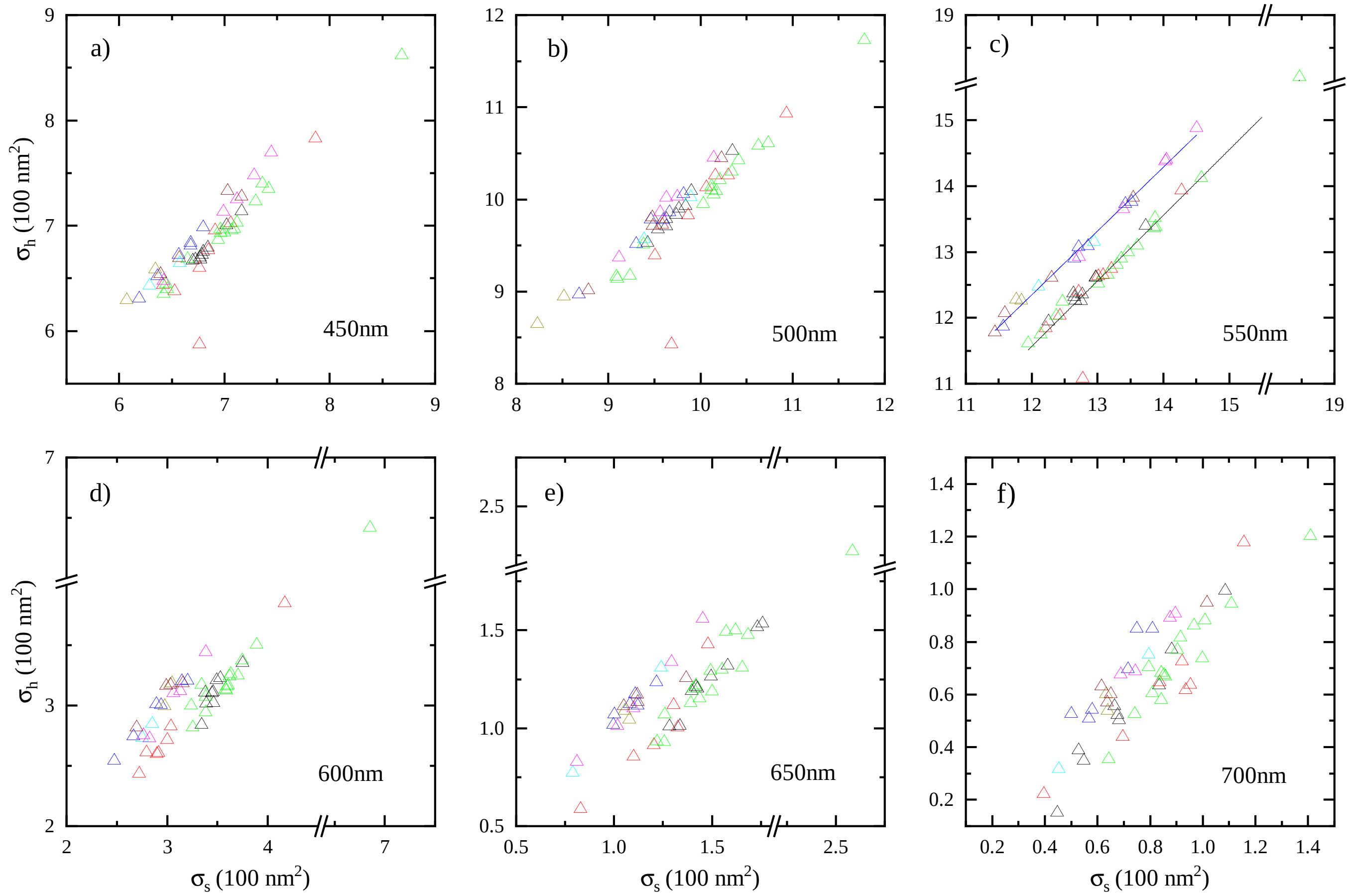

FIG. S5. Extinction cross-sections, σ_h, measured using the larger analysis radius, r_h as a function of the cross-sections, σ_s, measured using the standard analysis radius, r_s. Panels correspond to different filter center wavelengths Λ in the experiment as indicated. The symbol colour groups data by FOV. The lines in (c) are linear fits to the two visibly separated groups of data.

since less of the signal is integrated, such that a defocus in certain FOVs would result in a greater deviation compared to the well-focused cases. One can easily observe that while the same wavelength-dependent separation of FOV is apparent, certain FOVs (black, grey, red triangles) form an upper group already by $\Lambda = 450\,$nm.

Note, the data were taken with a sample reference shift of $s = 1550\,$nm. For our experimental setup and $\Lambda = [450\,\text{nm}, 500\,\text{nm}, 550\,\text{nm}, 600\,\text{nm}, 650\,\text{nm}, 700\,\text{nm}]$, we have $2r_s = [931, 1035, 1138, 1241, 1345, 1448]\,$nm, and $2r_h = [1117, 1241, 1366, 1490, 1614, 1738]\,$nm. For the longer wavelengths, $2r_h$ is comparable to s, which allows for some cross-talk. We also see that using r_h does lead to a lower IQR at all Λ, except for $\Lambda = 550\,$nm, where the defocus for some FOVs apparently was so large to extend the PSF tails beyond r_h. However the remaining discrepancy between the medians of the upper (five FOVs) and lower (three FOVs) groups of results for $\Lambda = 550\,$nm is still only about 4% using r_h.

Overall, assuming a fixed s and sample density, this examination indicates that in future, one could optimize the illumination NA to reduce r_i to the minimum necessary to collect the full extinction, and additionally reduce cross-talk between nearby NPs. Generally one could simply increase s and use a lower sample density. Furthermore, one can improve the focussing procedure or use automated focusing techniques. This discussion elucidates how the accuracy of extinction measurements within an experiment can affect the spread of retrieved sizes, beyond the measurement noise.

S5. EFFECT OF CORRECTIONS ON RETRIEVED NP MORPHOMETRY

In Fig. S7 and S8, we explore how the various corrections and minor improvements to the extinction and shape retrieval analyses affects the outcomes of the ON. Specifically, we look at the inclusion of lamp spectrum and camera sensitivity, use of different r_i in the extinction analysis, inclusion of $\sigma_{ext}(\gamma_R)$, and the correction of $\sigma_{ext}(\gamma_R)$ by a factor 0.97 (see Sec. IV A and Fig. 2). We find in Fig. S7(a-c) that the dominant effect on $\bar{S}$ are offsets, while the value of g for minimum $\bar{S}$ is less affected. The largest $\bar{S}$ is found for r_l, as expected from the larger defocussing artefacts. We find that when $\sigma_{ext}(\gamma_R)$ is not included, $\bar{S}$ reaches its lowest value across all cases, consistent with the reduced constraints on the fit, see also Sec. IV B. Including $\sigma_{ext}(\gamma_R)$, the lowest $\bar{S}$ is found using $0.97\sigma_{ext}(\gamma_R)$, in-

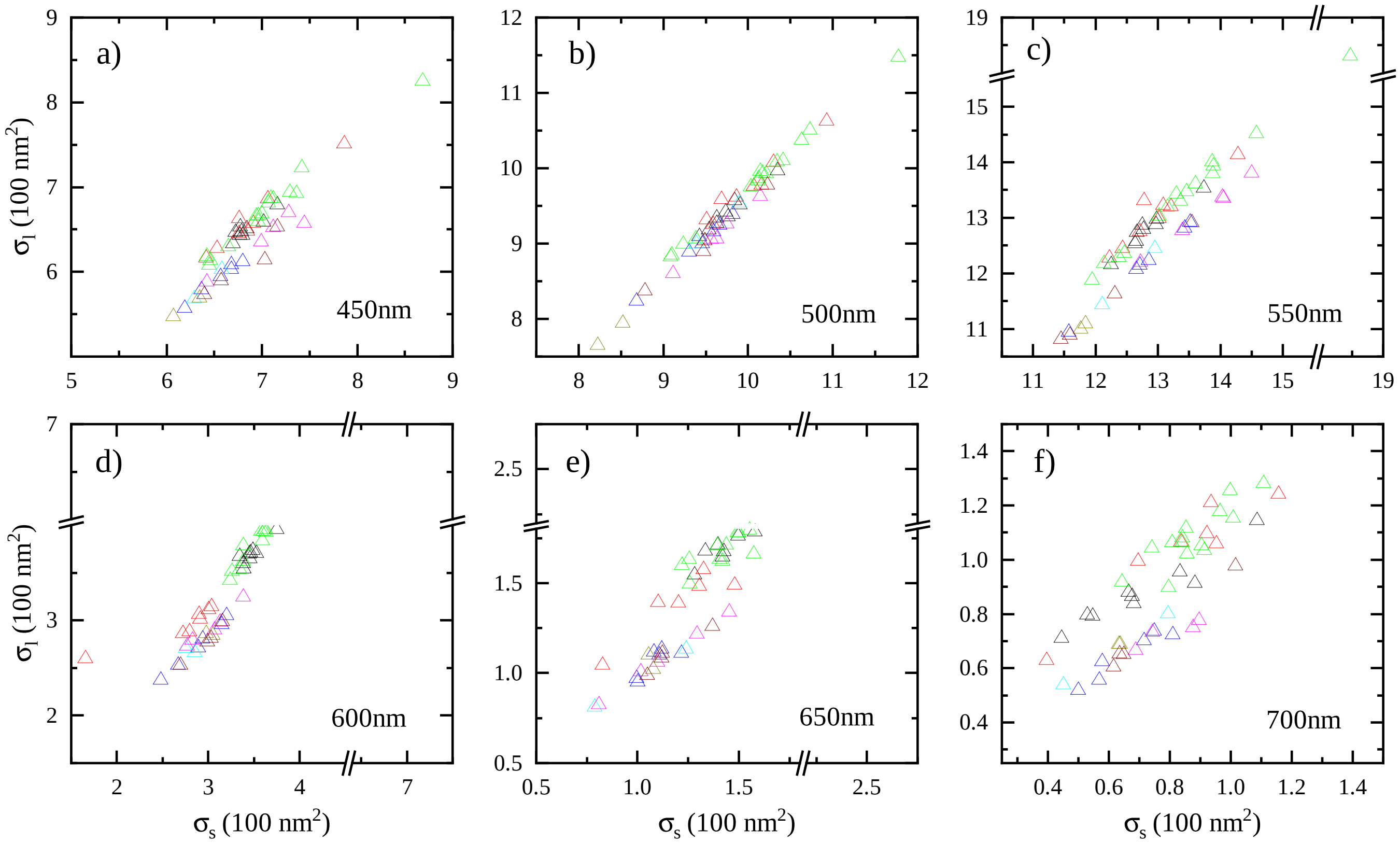

FIG. S6. As for Fig. S5, but using the smaller analysis radius, r_l.

cluding $w(\lambda)$, and using r_h in the extinction analysis. Across the analysis conditions we find $\bar{S}$ is smallest for McPeak, and largest for Olmon.

In Fig. S7(d-f) and Fig. S8 we show the dependence of the aspect ratios on g for the different permittivity datasets and analysis conditions. A considerably stronger dependence on g is observed for b/a than for c/a, with b/a increasing with g below the minimum of $\bar{S}$, and plateauing above. This can be understood by the broadening of the resonance, increasing the cross-sections at wavelengths longer than the LSPR, thus reducing the need for non-spherical shapes to shift the LSPR to the red.

We show the distributions of retrieved shapes and sizes for the different analysis conditions in Fig. S9. The condition +0.97γ_R,+r_h (panels g,h) provides the smallest spread and the roundest particles, closest to the shape seen in TEM of all conditions apart from those using $\varepsilon^s_\mathrm{NP}(\lambda)$ seen in Sec. IV B and Fig. 5. At the same time, it provides the smallest D_V, about 2 nm lower than TEM. This points towards a systematic error of the model. As discussed in Sec. IV B and Sec. V, a possible source are retardation effects, which are leading to a broadening by radiative damping and a red-shift compared to the static approximation used in our model.

S6. EFFECT OF MEDIUM REFRACTIVE INDEX ON RETRIEVED NP MORPHOMETRY

The effects of deviations of the refractive index of the NP environment from its nominal value $n_\mathrm{m} = 1.52$ on the morphometric retrieval are shown in Fig. S10 for OL with $g = 1.8$, and other analysis conditions the same as for the results in Fig. 4. We use changes of ± 0.02, which fully cover the range that might be expected from the spectral dispersion in glass or silicone oil over the probed wavelength range, or batch-to-batch material variations. We find that the dominant effect is on the retrieved shape, specifically on the AR c/b which changes by about twice the index change. Such a behaviour is consistent with the red-shift of the LSPR with increasing n_m, which is compensated by a reduced asymmetry-induced red-shift. These results indicate that the medium refractive index accuracy needs to be better than half the required aspect ratio accuracy. A slight change of the retrieved size is also seen, by about ∓ 0.25 nm for ± 0.02 index change.

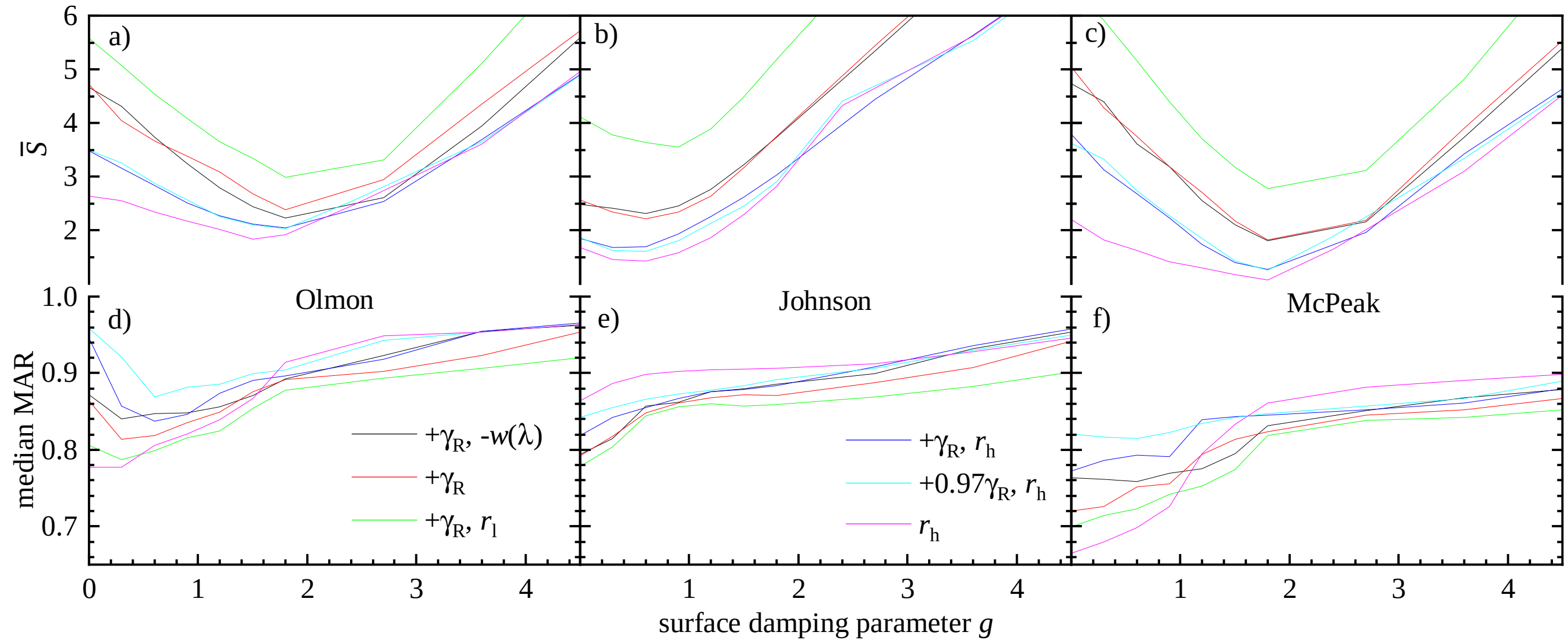

FIG. S7. Median error $\bar{S}$ and median MAR of ON analysis as function of surface damping parameter g, for permittivity datasets as indicated. Results for various analysis settings are shown, labelled as follows: $+\gamma_R$, $+0.97\,\gamma_R$ – inclusion of radially polarised measurements, unscaled or scaled by a factor of 0.97, respectively; -$w(\lambda)$ – no correction for the lamp spectrum and camera sensitivity; r_l, r_h – extinction data measured using the integration radius r_l or r_h, respectively.

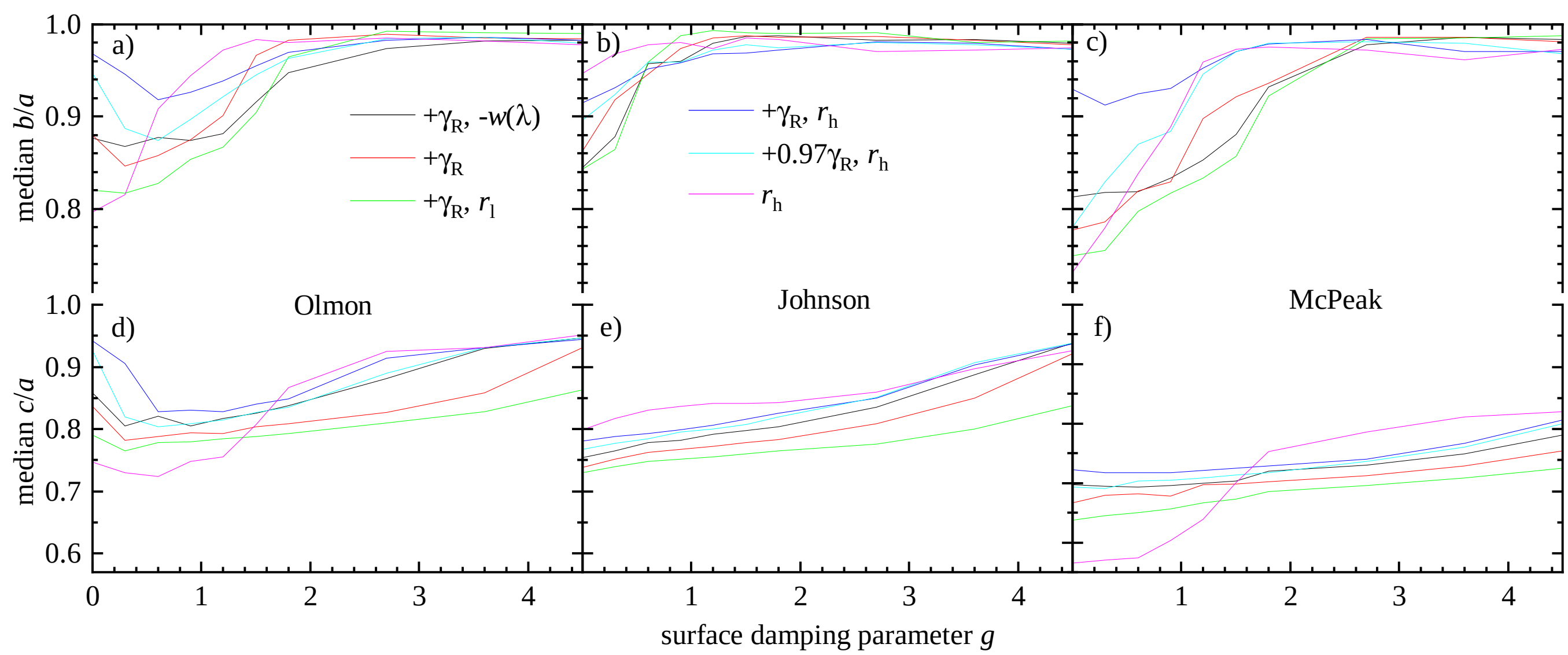

FIG. S8. As Fig. S7, but for the median of b/a and c/a.

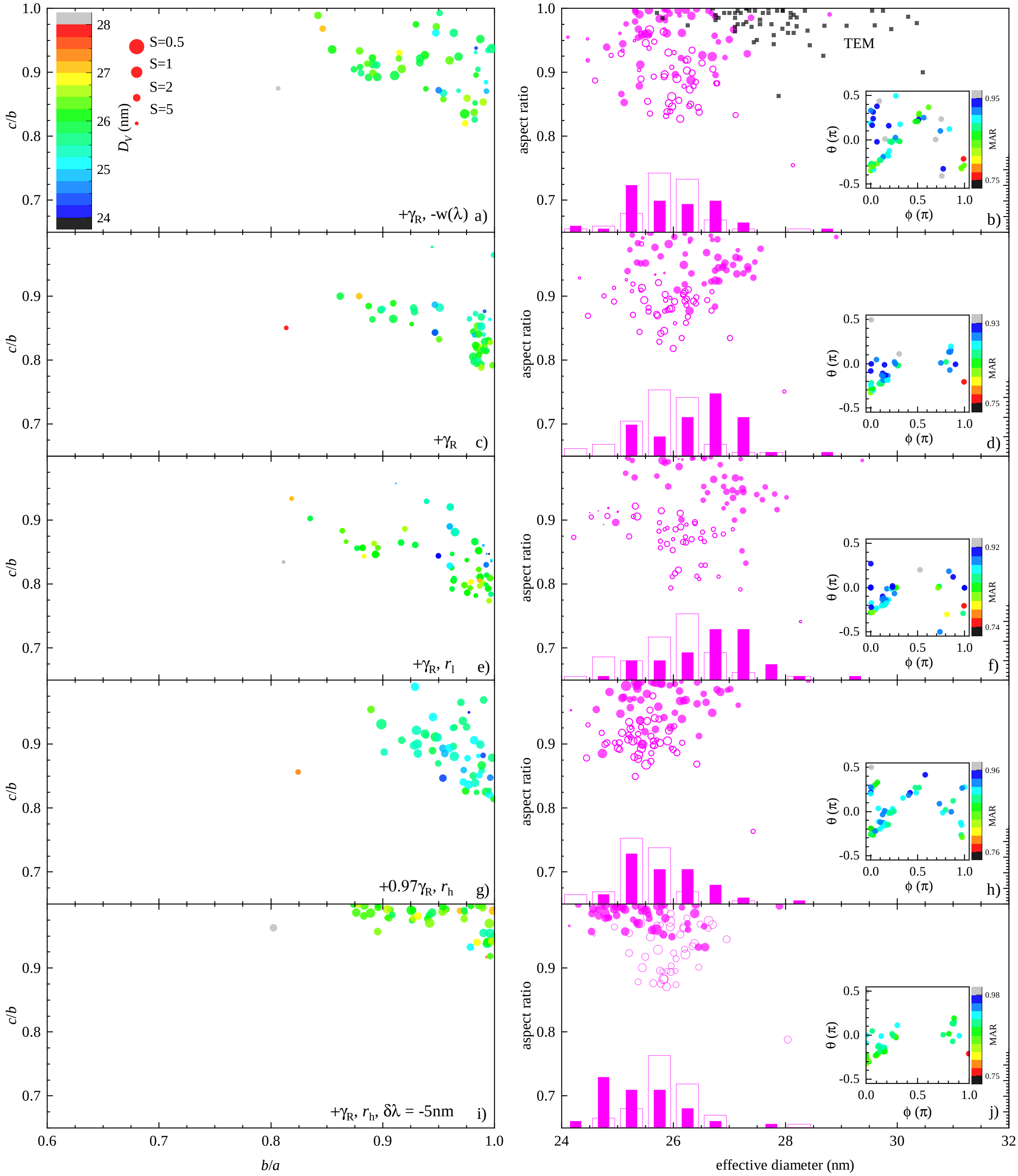

FIG. S9. Morphometric results for $N = 51$ nominally $D = 30$ nm UGNSs, for OL with $g = 1.8$, similar to Fig. 4, but for different analysis settings. Left column: fitted aspect ratios c/b versus b/a. The symbol size is proportional to $(1+S)^{-1}$ and indicates the error of the fit, as given in a), while the colour indicates the volume-equivalent diameter, D_V, as also given in a). Right column: MAR versus D_V (empty circles) and PAR versus D_p (filled circles), symbol sizes as in a). In b) the TEM data PAR versus D_p for the $N = 51$ UGNS is shown for reference. The histograms of D_V (empty) and D_p (filled) are also shown. The insets show the fitted angles, θ versus ϕ, with a symbol colour indicating the MAR as given in the scale. Using the designations introduced in Fig. S7, panels (a,b) refer to $+\gamma_\mathrm{R}$,$-w(\lambda)$; (c,d) to $+\gamma_\mathrm{R}$; (e,f) to $+\gamma_\mathrm{R}$,r_l; (g,h) to $+0.97\gamma_\mathrm{R}$,r_h; and (i,j) to γ_R,r_h, and $\delta\lambda = -5$ nm indicating a shift of the permittivity as discussed in Sec. S7.

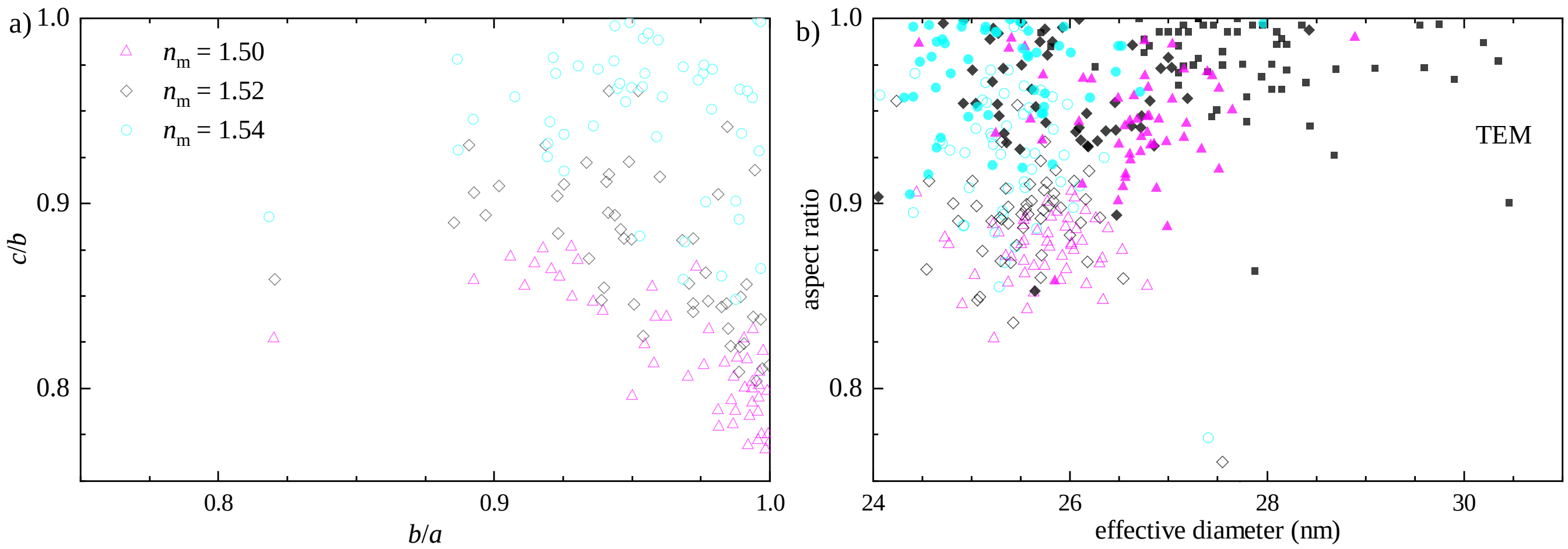

FIG. S10. Morphometric results for $N = 51$ nominally $D = 30\,\mathrm{nm}$ UGNSs, for OL with $g = 1.8$ similar to Fig. 4, but for varying medium index n_m. a) fitted aspect ratios c/b versus b/a. b) MAR versus D_V (empty symbols), and PAR versus D_p (filled symbols). The black filled squares indicate TEM measurements PAR versus D_p. Results are shown for the nominal index $n_\mathrm{m} = 1.52$ (black diamonds), a lower index $n_\mathrm{m} = 1.50$ (magenta triangles), and a higher index $n_\mathrm{m} = 1.54$ (cyan circles).

S7. EFFECT OF RETARDATION ON RETRIEVED NP MORPHOMETRY

Here, we consider if the small difference in aspect ratio and size between the retrieved morphometries and the TEM measurements is in part attributable to the fact that the dipole approximation does not take into account retardation effects. Specifically, in Fig. S11, we show the extinction spectra for a spherical NP of 27.94 nm diameter calculated using the Rayleigh-Gans theory, for all g used in the analysis, for each of the three permittivity datasets used in the main text. These are compared with calculations using Mie theory (dotted lines) at $g = 0$ and g yielding the minimum $\bar{S}$ for each of the three permittivity datasets. Importantly, we see that there is a small but noticeable red-shift in the peak of spectra calculated using Mie theory which includes retardation. Such a redshift can in the Rayleigh-Gans theory be achieved by increasing the shape asymmetry. To account for this red-shift without introducing a new model into the analysis, we shift the permittivity data by $\delta\lambda \in \{-4, -6, -8, -10\}$ nm, introducing $\varepsilon^s_{\rm NP}(\lambda) = \varepsilon_{\rm NP}(\lambda + \delta\lambda)$. We then perform the ON analysis using $\varepsilon^s_{\rm NP}$. To ensure we are not limited by the discussed small discrepancy between the linear and radial measurements of $\sigma_{\rm ext}$, we performed this additional analysis using the $0.97\times$ correction to the $\sigma_{\rm ext}(\gamma_{\rm R})$ data. To investigate the effect of the $0.97\times$ correction in the OL results shown in Fig. 5(a,b), we provide in Fig. S9(i,h) the corresponding results without that correction factor. Comparing the two results reveals that the factor has only a minor effect.

We examine the resulting dependence of $\bar{S}$ and the median MAR and PAR on g for the different $\delta\lambda$ in Fig. S12. OL and MP both show a minimum of $\bar{S}$ around $\delta\lambda = -4$ nm, while for JC only and increase is seen. MP shows a less clear dependence of MAR on g for the different $\delta\lambda$ compared to OL, but for all permittivities, larger $|\delta\lambda|$ provide an increased average roundness of the retrieved morphometries.

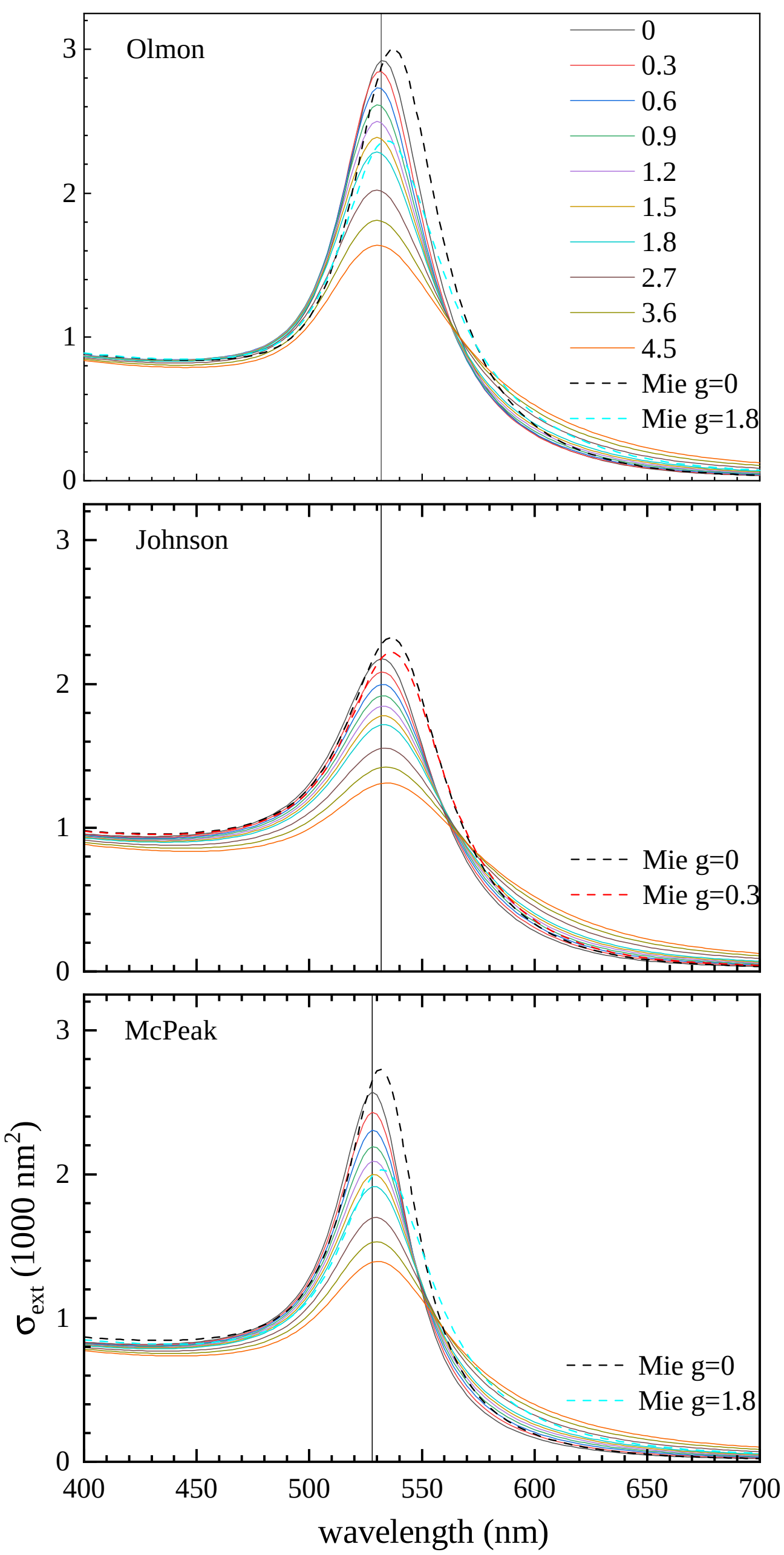

FIG. S11. Solid curves: $\sigma_{\rm ext}(\lambda)$ for a spherical NP of 27.94 nm diameter calculated using the Rayleigh-Gans theory for a range of g as indicated, for each of the three permittivity datasets described in the main text. Dotted curves: as above, but calculated using Mie theory at $g = 0$ and the g at which minimum $\bar{S}$ was obtained in the morphometric retrieval for each of the three permittivities ($g = (1.8, 0.3, 1.8)$ for OL, JC, and MP, respectively). Vertical lines indicate the peak position in $\sigma_{\rm ext}(\lambda)$ for $g = 0$ for the Rayleigh-Gans theory.

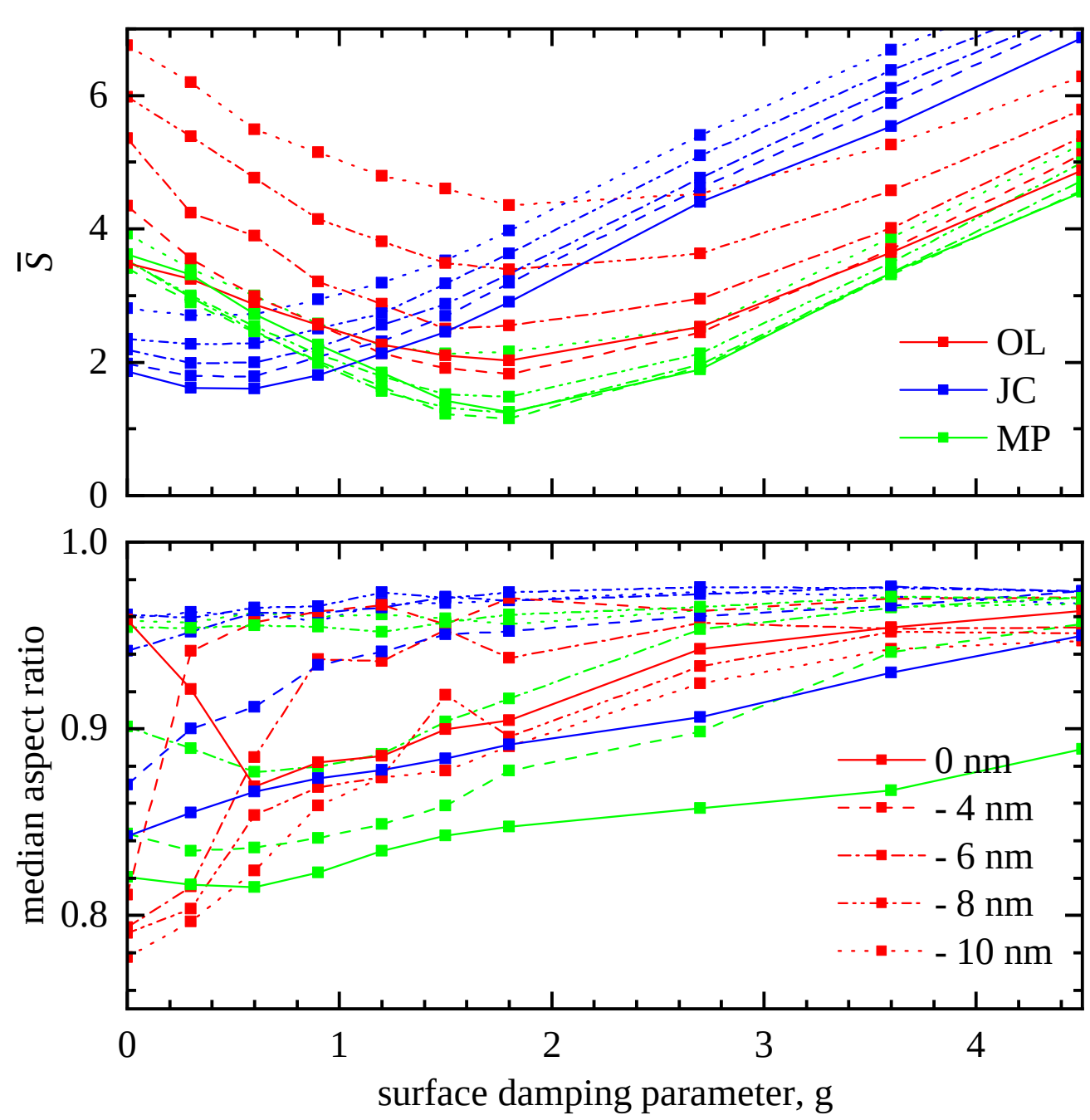

FIG. S12. $\bar{S}$ and median MAR for $N = 51$ UGNS of nominal $D = 30$ nm for varying surface damping parameter g as in Fig. 3, but using $\varepsilon^s_{\rm NP}(\lambda)$ for different $\varepsilon_{\rm NP}$ datasets indicated by color and wavelength shifts $\delta\lambda$ indicated by line type, as labelled.

TABLE S2. Summary table of important quantities and parameters.

Parameter name	Definition	Meaning
λ	-	Wavelength of light in vacuum
Λ	-	Center wavelengths of filters used in the experiment
g	-	Dimensionless scaling parameter of surface damping in the Drude model of permittivity, see Eq. 2
D	-	Nanoparticle (NP) diameter
V	-	NP volume
$\varepsilon_{\mathrm{NP}}$	-	Complex permittivity of the NP material
ε_{m}	-	Permittivity of the material surrounding the NP, assumed to be real and frequency-independent
k	$2\pi n_{\mathrm{m}}/\lambda$	Angular wavenumber in the medium of refractive index $n_{\mathrm{m}} = \sqrt{\varepsilon_{\mathrm{m}}}$
a, b, c	-	Semi-axes of the ellipsoidal NP defined such that $a > b > c$
x', y', z'	-	Cartesian axes aligned to the semi-axes of the ellipsoidal NP
α'_j	-	Diagonal elements of the NP polarisability in its own reference frame with $j \in \{1,2,3\}$
L_j	-	Depolarisation factors with $j \in \{1,2,3\}$
$\vec{E}'$	-	Vectorial electric field in the NP reference frame
$\vec{p}'$	-	Induced electric dipole moment in the NP reference frame
x, y, z	-	Cartesian axes of the laboratory reference frame
$\vec{E}$	-	Vectorial electric field in the laboratory frame
ϕ, θ, ψ	-	Angles of rotation about $\vec{e}_x$, $\vec{e}_y$, $\vec{e}_z$, respectively
MAR	$\sqrt{bc}/a$	Mean aspect ratio
a', b'	-	Semi-axes lengths in the xy-plane of the ellipsoid projected along the z axis
PAR	b'/a'	Projected aspect ratio
D_V	$2\sqrt[3]{abc}$	Volume-equivalent diameter
D_{p}	$2\sqrt{a'b'}$	Projected diameter
I_{inc}	-	Incident intensity
P_{abs}	-	Power absorbed from the incident field
P_{sca}	-	Power scattered from the incident field
σ_{abs}	$P_{\mathrm{abs}}/I_{\mathrm{inc}}$	Absorption cross-section
σ_{sca}	$P_{\mathrm{sca}}/I_{\mathrm{inc}}$	Scattering cross-section
σ_{ext}	$\sigma_{\mathrm{abs}}+\sigma_{\mathrm{sca}}$	Extinction cross-section
$\hat{\sigma}_{\mathrm{ext}}$	-	Shot-noise-limited standard deviation in the measured σ_{ext}
$\sigma_\Lambda(\gamma_{\mathrm{P}})$	-	σ_{ext} measured using the filter with center wavelength, Λ, and the linear polariser in the illumination
$\sigma_\Lambda(\gamma_{\mathrm{R}})$	-	σ_{ext} measured using the filter with center wavelength, Λ, and the radial polariser in the illumination
$\bar{\sigma}_\Lambda$	-	Mean of σ_Λ over the NPs
$\hat{\sigma}_\Lambda$	-	Standard deviation of σ_Λ over the NPs
$M\{.\}$	-	Median of the quantity in brackets over the NPs
γ_{P}	-	Excitation polarisation angle, indicating the use of linearly polarised illumination
γ_{R}	-	Virtual excitation polarisation angle indicating the use of radially polarised illumination
r_{i}	$3\lambda/(2\mathrm{NA})$	Reference integration radius used in obtaining measured σ_{ext} from image data
r_{s}	r_{i}	Standard value of the integration radius employed in the analysis
r_{h}	$1.2\,r_{\mathrm{i}}$	Higher value of the integration radius employed in the analysis
r_{l}	$0.8\,r_{\mathrm{i}}$	Lower value of the integration radius employed in the analysis
η	0.646	Fraction of scattered light not collected by the objective, and hence contributing to the measured σ_{ext}
S	-	Normalized error between measured and modelled extinction for a NP, minimised during the ON analysis
$\bar{S}$	$\sqrt{M\{S^2\}}$	The median of S taken over the NPs
$\varepsilon^s_{\mathrm{NP}}(\lambda)$	$\varepsilon_{\mathrm{NP}}(\lambda+\delta\lambda)$	$\varepsilon_{\mathrm{NP}}$ shifted by a small amount, $\delta\lambda$ to approximate the effects of retardation observed in Mie theory

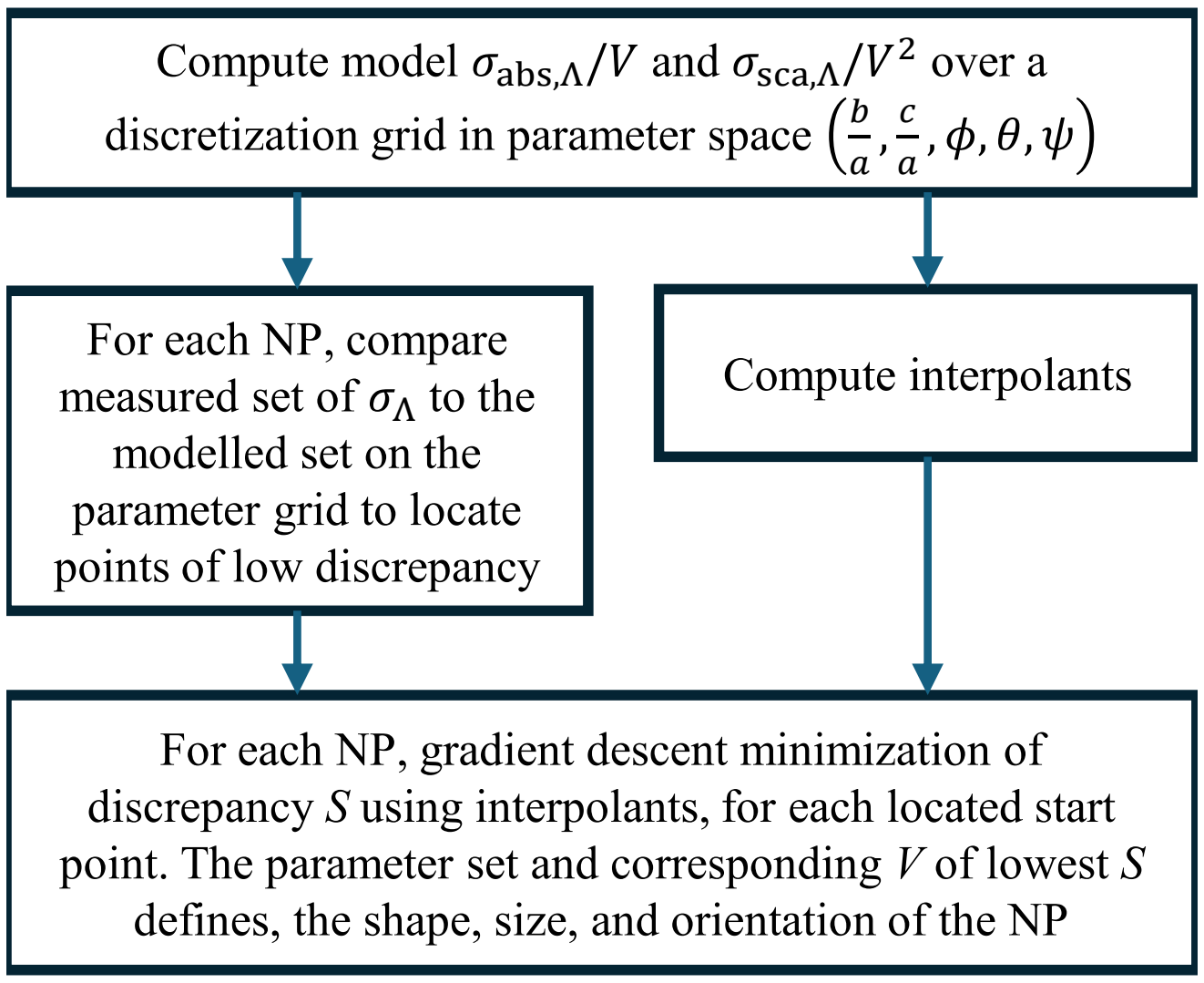

FIG. S13. Schematic of the analysis flow to determine the NP semi axes a, b, c and orientation ϕ, θ, ψ from the measured extinction cross-sections, minimising S (see Eq. 11).

[1] L. Payne, *Optical extinction and coherent multiphoton micro-spectroscopy of single nanoparticles*, Ph.D. thesis, Cardiff University (2016).

[2] R. Hartley and A. Zisserman, *Multiple view geometry in computer vision* (Cambridge university press, 2003).

[3] R. J. Yamartino, "A Comparison of Several "Single-Pass" Estimators of the Standard Deviation of Wind Direction," J. Clim. Appl. Meteorol. **23**, 1362–1366 (1984).

[4] L. Payne, A. Zilli, Y. Wang, W. Langbein, and P. Borri, "Quantitative high-throughput optical sizing of individual colloidal nanoparticles by wide-field imaging extinction microscopy," in *Proc. SPIE 10892, Colloidal Nanoparticles for Biomedical Applications XIV*, Vol. 10892 (2019) pp. Colloidal Nanoparticles for Biomedical Applications XIV, 108920J.
